# Enhanced statistical sampling reveals microscopic complexity in the talin mechanosensor folding energy landscape

**DOI:** 10.1038/s41567-022-01808-4

**Published:** 2022-11-07

**Authors:** Rafael Tapia-Rojo, Marc Mora, Stephanie Board, Jane Walker, Rajaa Boujemaa-Paterski, Ohad Medalia, Sergi Garcia-Manyes

**Affiliations:** 1 Single Molecule Mechanobiology Laboratory, The Francis Crick Institute, 1 Midland Road, London NW1 1AT, London, UK; 2 Department of Physics, Randall Centre for Cell and Molecular Biophysics, Centre for the Physical Science of Life and London Centre for Nanotechnology, King’s College London, Strand, WC2R 2LS London, United Kingdom; 3 Department of Biochemistry, Zurich University, Winterhurerstrasse 190, CH-8057, Zurich, Switzerland

## Abstract

Statistical mechanics can describe the major conformational ensembles determining the equilibrium free-energy landscape of a folding protein. The challenge is to capture the full repertoire of low-occurrence conformations separated by high kinetic barriers that define complex landscapes. Computationally, enhanced sampling methods accelerate the exploration of molecular rare events. However, accessing the entire protein’s conformational space in equilibrium experiments requires technological developments to enable extended observation times. We developed single-molecule magnetic tweezers to capture over a million individual transitions as a single talin protein unfolds and refolds under force in equilibrium. When observed at classically-probed timescales, talin folds in an apparently uncomplicated two-state manner. As the sampling time extends from minutes to days, the underlying energy landscape exhibits gradually larger signatures of complexity, involving a finite number of well-defined rare conformations. A fluctuation analysis allows us to propose plausible structures of each low-probability conformational state. The physiological relevance of each distinct conformation can be connected to the binding of the cytoskeletal protein vinculin, suggesting an extra layer of complexity in talin-mediated mechanotransduction. More generally, our experiments directly test the fundamental notion that equilibrium dynamics depend on the observation timescale.

Proteins must fold into their native state to be functional. How proteins search and attain their native structure as they emerge from the ribosome, and when they repeatedly unfold and refold in their physiological setting, remains a pivotal question in biology^1^. While the protein folding field has witnessed outstanding progress^2^, massively helped by the prolific combination of experiments of varying nature with theoretical modelling and computational tools, our knowledge is still far from complete^3^. The classical view of folding was heavily grounded in the relatively simple concept of a sequential pathway followed by the unfolded polypeptide during its journey to the native state through specific landmark intermediates. Later, the new view of protein folding offered a statistical-mechanics conceptual framework involving multi-parallel and disordered folding trajectories (as opposed to a single, univocal pathway) visiting ensembles of intermediate conformations^4–8^. To support this view, the funnel energy landscape description has enormously helped picturing the diffusive evolution of an unfolded chain through a progressively narrower conformational space that eventually leads to the low-energy native state basin^5,9,10^. To facilitate the search and ensure that the folded structure is reachable on biologically-relevant timescales, nature has evolved proteins to generally satisfy the minimal frustration principle, implying that, if (at all) stuck in conformational intermediates, these will be short-lived, implying that the underpinning barriers must be small enough to be overcome by thermal fluctuations^11–13^.

Protein folding biochemistry experiments conducted in the bulk excel at capturing global averages, hallmarking ensembles of conformations. Similarly, classical statistical mechanics models have helped identify the physical principles underlying protein folding, often compromising microscopic details. By contrast, recent computationally expensive molecular dynamics (MD)-based approaches achieve an atomic level of detail on the folding of a single protein, at the detriment of severely reducing the number of available trajectories^14^. Single-molecule experiments on an individual protein exposed to force enable direct observation of its conformational dynamics during an individual (un)folding trajectory along a one-dimensional (1D) projection of the full landscape^15^ onto the protein’s end-to-end length reaction coordinate, which appears to be a good folding descriptor even for relatively complex systems^16^. Equilibrium single protein nanomechanical experiments rely on maintaining the applied force fixed while the protein spontaneously hops between different conformations, driven by thermal fluctuations^17^. The average timescales associated to each transition provide a direct description of the (un)folding dynamics characterising each visited state^18–26^. Despite offering an unbiased method to probe the protein’s conformational space, force spectroscopy equilibrium experiments have been hitherto severely limited by the experimentally achievable timescales, often precluding sampling of low-probability conformations that require surmounting high kinetic barriers^27^, effectively rendering the involved state(s) difficult to quantify experimentally.

Reconstructing the protein’s folding energy landscape requires unambiguous identification of all the accessible conformations and the position and height of energy barriers separating them. Under equilibrium conditions, this is typically achieved by capturing a sufficiently large number of independent folding trajectories, ensuring a complete and accurate sampling. If the protein exhibits uncomplicated folding, the most probable folding routes might be easily captured within the timescales set by the most common measuring techniques. However, if the folding energy surface is complex, containing multiple non-native metastable states, the observation window needs to significantly expand in order to detect the low-probability events. This poses a real challenge to most standard measuring —experimental and computational— techniques. From the computational perspective, the field of molecular dynamics has devoted significant efforts to develop enhanced-sampling methods (*e.g*. metadynamics^28^, milestoning^29^, or parallel tempering^30^, to name a few) to artificially bias the dynamics, hence dilating the accessible timescales. However, equilibrium experiments must rely on a ‘brute force’ approach that unavoidably requires to experimentally capture thousands (or ideally, millions) of independent (un)folding transitions in an individual protein, posing significant pressure to overcome present experimental limitations.

Here, we designed a novel simplified magnetic tweezers that, by replacing the classically used vertically-displacing pair of magnets with a magnetic tape head^31^, provides significantly improved stability, allowing us to measure the dynamics of single proteins for days in a driftless manner (Fig 1A and Fig. S1). Akin to optical tweezers^26,32,33^, the intrinsic passive clamp conditions of magnetic tweezers provide the natural statistical ensemble for studying protein nanomechanics in equilibrium. Using this approach, we studied the dynamics under force of an individual protein consisting of a single monomer of the talin’s R3 domain^34^ harbouring the IVVI mutation (R3^IVVI^) —which increases the mechanical stability of the R3^wt^ domain^35,36^ and slows down nuclear mechanotransduction in cells^37^— bracketed between two stiff titin Ig32 domains (spacers) at each side (Fig. 1A)^38^. When held at the coexistence force *F*_0.5_ (~8.5 pN, in agreement with previous observations^36^) for 500 s (Fig. 1B, top panel), the R3^IVVI^ protein hops between the native and unfolded states in equilibrium. However, as the measuring time is increased by one (Fig. 1B, middle panel) or two (Fig. 1B, lower panel) orders of magnitude, R3^IVVI^ visits new, low-probability states that can be easily identified by both a change in the (un)folding step size and in their intrinsic dynamics with respect to the common two-state scenario (Fig. 1C. ***i***). After systematic analysis of multiple trajectories accumulating over 500,000 seconds of protein dynamics, 5 regions of distinct *rare* states can be regularly singled out (Fig. 1C and Extended Data Fig. 1, ***ii-vi***). While some of these states are populated by a single conformation (namely ***iii***, ***iv***), other states (***ii**, **v***, and ***vi***) are composed of multiple interconverting conformations. The identification of these low-probability states was independent of the molecular handles flanking the R3^IVVI^ domain (Fig. S2). Taken together, these experiments demonstrate that, on timescales spanning from seconds to several minutes, R3^IVVI^ typically samples a local equilibrium, displaying an uncomplicated two-state folding scenario. As the unfolding/refolding equilibrium dynamics is observed for significantly larger timescales (hours to days), R3^IVVI^ is able to progressively overcome larger energy barriers and explore previously inaccessible regions of the protein’s conformational space (Fig. 1D). Interestingly, while falling into one of these uncommon states (Fig. 1C, ***ii-vi***) is a low-probability event (implying a slow kinetic on-rate), escaping from that given state can be similarly difficult (slow off-rate). This implies that R3^IVVI^ can be trapped in one such low-probability state for extended periods of time (cf. Fig 1B, lower panel), indicating that (most of) these conformations are thermodynamically stable; however, in all cases, R3^IVVI^ eventually escapes these rare states and recovers its native folding dynamics ***i***(Fig. 1C).

To probe the effect of force on the reversibility of these rare states (and to exclude the possibility that R3^IVVI^ has irreversibly misfolded due to *e.g*., protein oxidation^39,40^), we designed a force-pulse protocol aimed at rescuing the protein from the trapped conformations (Fig. 2). In the individual trajectory shown in Fig. 2A, the dynamically (un)folding protein spontaneously falls into one such rare state (***v*** in this case) when held at *F*0.5. We then quenched the force down to 4 pN for a long period of time (2 hours). The protein remained trapped in state ***v***, as confirmed by the lack of two-state folding when the force was increased back to *F*_0.5_. However, after a brief pulse at a higher force (15 pN) that drove the protein to the unfolded form, the two-state dynamics between the folded and unfolded conformations was recovered, effectively demonstrating that R3^IVVI^ had escaped from the low probability region. This experiment shows that the application of a high force pulse brings the system back to its canonical folding route. We next measured the rescue probability as the force varied within the 4 pN-20 pN range (Fig. 2B), concluding that the destabilization of the trapped conformations is highly force-dependent. While low forces (4 pN) help trap talin in the rare states (implying that the escape barrier is simply too high), high forces (10-20 pN) trigger the disruption of the enthalpic interactions that hold R3^IVVI^ in conformationally rare structures, effectively unfolding and resetting the protein’s capability to engage in the main two-state folding pathway. The force-dependent escape for each of the other low-probability states follows a similar trend (Extended Data Fig. S3).

In an effort to elucidate the microscopic origin of each state (***ii-vi***), we next set out to examine them independently. When held at the coexistence force (*F*_0.5_) for an average of 1,000 s, R3^IVVI^ stochastically enters state (***ii***), which exhibits the same step size between the folded and unfolded conformations as the regular (***i***) pathway, yet its mechanical stability is shifted to lower values. This implies that, at *F*_0.5_, the protein suddenly lies mostly in its unfolded conformation, exhibiting only brief excursions to the folded form (Fig. S3). We conjectured that this conformational change might be explained by a cis-trans isomerization of proline 881 (the only proline within the R3 fold, Fig. 3A-C)^21,41^. This was confirmed by studying the dynamics of a proline-to-glycine P881G R3^IVVI^ mutant protein (Fig. 3D), recapitulating the force-dependent dynamics of the mechanically labile trans isomer of the R3^IVVI^ protein (Fig. 3E and Fig. S4). Noteworthy, examination of the folding dynamics of the R3^IVVI^ P881G mutant also revealed the presence of all the low-probability rare states (***iii***, ***iv***, ***v*** and ***vi***) previously described, except state ***ii*** (Fig. S5). Crucially, the rate of cis-trans conversion in R3^IVVI^ is accelerated by force (Fig. 3F) and closely follows the unfolding probability (Fig. 3E), suggesting that isomerization of P881 is favoured when talin is unfolded.

While state (***ii***) underpins a conformational transition that changes the force-dependent (un)folding dynamics of the protein, states (***iii-vi***) are all characterized by intermediate molecular extensions that do not correspond to either the folded or the unfolded forms of the protein. A closer inspection of each of the states also reveals a different level of conformational fluctuations (or “noise”) fingerprinting each state (Fig. 1D). The ability to work at low pulling forces enables magnetic tweezers to capture the natural fluctuation modes of the protein under force. We recently demonstrated that the protein’s stiffness scales with the pulling force according to the sequence-independent freely-jointed chain model^38^. This implies that the end-to-end fluctuations <Δσ^2^_i_(*t*)> are a direct hallmark of the contour length increment (Δ*L*_C_) of the unstructured polypeptide released upon unfolding at a given force. Given that two conformations fingerprinted by the same end-to-end length <Δ*z_i_*> could potentially exhibit distinct fluctuations if they contained a different extent of unstructured regions (Fig. 4A), we conjectured that this variance analysis method might be a useful tool in providing plausible additional structural hints for each conformation encountered in the folding conformational space.

We applied a threshold algorithm to automatically detect each conformation (labelled by capital letters, Fig. 4) within each particular state (labelled by roman numerals, ***i-vi***) as characterized by their end-to-end extension <Δ*z*_i_(*t*)>, see SI methods and Fig. S6. From the idealized trajectories, we quantified the molecular fluctuations <Δσ^2^_i_(*t*)> of each conformation as σ^2^_i_(*t*)= <*z*_i_(*t*)>^2^ - <*z*_i_(*t*)^2^>, with the native conformation as reference. Assuming that secondary structure elements do not significantly contribute to the molecular fluctuations, the unstructured contour length Δ*L*_C,i_ of a particular conformation can be estimated from the freely-jointed chain model of polymer elasticity (which provides a convenient and natural *z*(*F*) relationship) as:

$$\text{Δ}L_{C,i}=\frac{\langle \text{Δ}\sigma_{i}^{2}\rangle }{l_{K}\left[1-\coth^{2}\left(\frac{Fl_{K}}{kT}\right)+{\left(\frac{kT}{Fl_{K}}\right)}^{2}\right]}$$

Where *l*_K_=1.1 nm is the Kuhn length, and *kT*=4.11 pN nm. Normalizing Δ*L*_C,i_ by the contour length increment of R3^IVVI^ (Δ*L*_C,U_=42.5 nm), one can estimate for each specific conformation the fraction of unstructured protein sequence.

We next used this analysis to provide a first structural guess of the different protein conformations underpinning each distinct region. In a first attempt to simplify the number of possible resulting structures, and based on both computational^14^ and experimental^42–44^ evidences showing that α-helix structures are locally formed very fast (on the microsecond timescale), followed by topological reorganization, structure consolidation and final packing of the protein^45^, we conjectured that the 4 helixes of talin are likely to form independently, reminiscent of the “foldon”^1^ concept, and that it is the interaction between the different helixes^46^ that determines talin’s folding fate. When applied to state ***iii*** (Fig. 4B), we observed that, while its end-to-end length lies between that of the unfolded and folded conformations, it exhibits fluctuations akin to those of the folded form, indicating that this conformation lacks unstructured segments. A plausible structure compatible with these measurements is shown in Fig. 4B. In contrast to the rather short-lived (~7 s) nature of state ***iii*** (implying an associated fast escape rate), state ***iv*** is comparatively more populated and stable (~100 s), and is topologically fingerprinted by a short (~3 nm) reduction in length with respect to the unfolded conformation. However, the concomitant fluctuations are massively reduced, corresponding to an unstructured contour length halfway (~22 nm) between the unfolded and native forms. Incidentally, state ***iv*** is reminiscent of the vinculin-bound conformation of R3^IVVI^, which involves a coil-to-helix transition in both vinculin-binding sites^47^, compatible with our topological and fluctuation measurements (Fig. S7). It is hence interesting to observe that state ***iv*** is already available in the full conformational space of R3^IVVI^, but that vinculin binding largely favors and stabilizes this conformation.

In contrast to states ***iii***-***iv***, which exhibit a unique conformation reminiscent of a single free energy basin, states ***v*** and ***vi*** involve internal dynamics, implying that R3^IVVI^ can interconvert between several conformations or sub-states (namely 3 conformations in ***v*** and 2 in ***vi***) separated by barriers that are much lower than both the entry and exit barriers. In other words, states ***v*** and ***vi*** exhibit relatively fast internal dynamics, yet their kinetics for both accessing and escaping is seemingly slow, implying that, once visited, both states are long-lived and kinetically trapped. To demonstrate that the internal dynamics in states (***v***) and (***vi***) correspond to transitions between distinct protein conformations, we probed their individual force-dependency, which follows the expected behaviour (increasing forces populate the higher extension states). Interestingly, both states (***v***) and (***vi***) exhibit a broad force-dependency (Extended Data Fig. 3, 4, 5, and 6) — in particular, state ***v*** spans ~8 pN to fully reach the most extended conformation —, in stark contrast with the steep force-dependency (~1.5 pN) of the native (un)folding dynamics (***i***) of R3^IVVI 36^, underpinning the mechanosensory behavior of talin. Noteworthy, each conformation forming states ***v*** and ***vi*** can be clearly singled out by their unique change in overall contour length (Δ*z_i_*) and molecular fluctuations (Δσi^2^), enabling us to hypothesize a set of plausible structures compatible with each conformation (Fig. 4D, E). Extended Data Table 1 summarises the configurational properties of each distinct state (***i-vi***), which, combined, define the full conformational space of the talin R3^IVVI^ domain.

Our long-equilibrium measurements show that the conformational space of the talin R3^IVVI^ domain can be partitioned into six distinct states (***i-vi**)*, each of them being characterized by conformations that can be uniquely fingerprinted by their end-to-end length and fluctuation signatures (Extended Data Table 1). Measuring the transition times between those conformations enabled us to map R3^IVVI^’s equilibrium landscape as a kinetic network reconstructed at the coexistence force *F*_0.5_. We systematically analyzed several independent equilibrium trajectories (*N*=13) at *F*_0.5_ lasting a sufficiently long time (>10,000 s) to ensure we obtained an unbiased, statistically robust representation, and established a hierarchy of states in terms of their probability of occurrence (on-rate) and probability of escape (off-rate), together with the interconversion rates between sub-states (Fig. 5 and Extended Data Table 2). Of note, all states except ***iii*** are linked only to the unfolded conformation, suggesting that lateral connections between rare states (*i.e*., by-passing the canonical two-state folding route) are vanishingly small. In this sense, while state ***iii*** can be interpreted as a classic intermediate (Fig. S8), states ***iv-vi*** represent failed folding excursions, where the unfolded protein diffuses over a large barrier that reveals a previously undiscovered region of the free energy landscape.

Combined, these observations naturally lead to the question of whether other (or all) model mechanical proteins might also depart from the classically assumed two-state (un)folding kinetics provided the experimental measuring time window is massively increased. We hence conducted similar experiments to measure the dynamics of the bacterial Protein L protein, which has been classically reported to also fold in an all-or-none manner^31^, over extended timescales as long as 58 hours (Extended Data Fig. 7). Remarkably, unlike talin R3^IVVI^, and despite its slower kinetics, protein L does not visit any state different from the native and unfolded protein forms, suggestive of an uncomplicated two-state energy landscape that remains smooth even after extended measuring time. Consequently, talin seems to be rather unique in that its complexity readily emerges as the experimental measuring time increases. We cannot exclude that even more states with lower probability (*i.e*., higher barriers) could be unveiled if the observation time were increased up to weeks or even months, but our experimentally achievable measurements to date (up to several days) display a robust well-defined number of conformationally distinct states that repeat themselves over time within the same protein and within different proteins over time.

We last queried about the putative biological relevance of the low-probability states that we identified. Functionally, the mechanical unfolding of talin exposes previously cryptic binding sites that enable full-length vinculin binding^47,48^ (Fig. 6) thereby strengthening its interaction with the actomyosin cytoskeleton. Hence, talin’s mechanical function, and namely that of its R3 domain, is intimately related to its capacity to unfold and refold under force and to ensure efficient vinculin binding. Consequently, we set out to probe which of the low-probability states were functionally able to bind vinculin. At 20 nM, vinculin binds the unfolded R3^IVVI^ conformation in a few seconds (~30 s on average), and the binding event can be unambiguously hallmarked by a reduction of R3^IVVI^’s extension by ~3 nm and the interruption of the folding dynamics^35,47^(Fig. 6B). However, when adding 20 nM vinculin after R3^IVVI^ spontaneously fell onto state ***v*** (Fig. 6C), the three-level dynamics hallmark of state ***v*** remained unchanged over an hour, suggesting that vinculin cannot bind when talin is trapped in this conformational mode. Only after applying a 40 pN pulse to rescue R3^IVVI^ back to its native folding dynamics, did we observe the distinctive vinculin-binding fingerprint, indicative that active vinculin was present in the solution. In summary, this experiment revealed that state ***v*** is non-functional from the perspective of its ability to bind full-length vinculin. Repeating this experiment for all the other states (Fig. S9, S10, and S11) concludes that vinculin binds to state ***i***, ***ii*** and ***iv*** (in fact this is indistinguishable because we suggest that this is the same state stabilized upon vinculin binding). By contrast, vinculin cannot bind states ***v*** and ***vi*** (state ***iii*** is so short-lived that we could not specifically test its ability to bind vinculin). Such a functional screening enables us to distinguish and categorize the different states according to their potential ability to retain talin’s main biological function. It is interesting to observe a rough correlation between their probability of occurrence and their functionality; while those ‘more common’ rare states (***i***, ***ii***, and ***iv***) can successfully bind talin, those less accessible (states ***v*** and ***vi***) cannot. In that sense, states ***v*** and ***vi*** might be considered misfolded states in the more classical definition (*i.e*., ‘not able to perform function’) yet these are not irreversible, since albeit being extremely stable and long-lived, they can eventually spontaneously resolve over long timescales.

In conclusion, by extending our equilibrium single-molecule experiments to capture hours-long trajectories, involving over a million individual unfolding and refolding transitions, we have uncovered signatures of unprecedented complexity in the equilibrium (un)folding energy landscape of the talin R3^IVVI^ mechanosensor —a model mechanical protein that was thus far assumed to follow uncomplicated two-state folding dynamics^36,49^. Increasing the measuring time enabled detection of low-probability states and rare conformational *cul-de-sacs*, alongside their relationship to function. We suggest that, in the most frequent folding scenario (***i***), the 4 helices forming the talin R3^IVVI^ domain fold cooperatively on our experimental time resolution (~1-2 ms for ~1,500 Hz sampling rate), thus resulting in an effective two-state folding energy landscape (Extended Data Fig. 8). We speculate that the non-native arrangements between the 4 helixes are likely to underpin most of the rare conformations that we capture (***iii***, ***iv***, ***v***, ***vi***). Thanks to new fluctuation variance analysis, dwelling on the fact that only unstructured regions of the protein significantly contribute to its fluctuation, we could begin to provide structural suggestions to each trapped conformation. Guiding our structural predictions on the ability of each helix (foldon) to fold as an independent folding entity gives rise to structures that are well compatible with our contour length measurements and end-to-end fluctuations, however, we cannot of course exclude other possible structures. More generally, our experiments integrate some ingredients from both the old and new views of protein folding. On the one hand, the notion that enhanced statistical sampling enables to overcome generally inaccessible barriers is reminiscent of the landscape portrait that encapsulates the statistical mechanics description of protein folding. On the other hand, the ability of capturing precise and well-defined protein conformations also resonates with the old view – although, except for ***iii***, which displays excursions (albeit rare) to the native form, those rare conformations that we capture are not necessarily precursors of the natively folded conformation but rather closer to the classically defined off-pathway intermediates^50^. More broadly, our experiments focusing on the thermodynamic equilibrium states forming the conformational space of a dynamically folding protein complement recent elegant single molecule experiments concentrating on the transition paths between well-defined protein conformations^16,33^. While our experiments are conducted at room temperature, we speculate that the interconversion dynamics across the conformational space will be modulated by temperature ^51^. Finally, from a functional perspective, it is difficult to gauge the biological relevance of the timescales required to access these low-frequency states. However, given that the probed forces are within the physiological range^52^, that it takes ~700-1,500 seconds for talin to dissociate from focal adhesions^53^ and that focal adhesion maturation can last up to tenths of minutes^54–56^, it is tempting to speculate that the low-probability states that we detected in talin R3 might be populated during the time-course of focal adhesion assembling and maturation. It is hence conceivable that those talin molecules trapped in rare states that cannot bind vinculin, albeit stretched, might not be able to effectively engage with the actin cytoskeleton^57^, thereby likely providing extra broadening in the timescales involved in cellular mechanotransduction.

## Methods

### Protein Expression and Purification

#### Polyprotein engineering for Magnetic Tweezers experiments

To construct the (Ig32)_2_- (R3^IVVI^)-(Ig32)_2_ and (Ig32)_2_-(R3^IVVI^ P881G)-(Ig32)_2_ polyproteins, (Ig32)_2_-(R3^IVVI^)-(Ig32)_2_ DNA was synthesised by GeneArt (Thermo Fisher Scientific). This was designed with BamHI-KpnI sites for insertion into a modified pFN18a plasmid and contained BspEI and NheI sites around the R3^IVVI^ sequence to allow replacement sequences to be inserted. The pFN18a plasmid was engineered with the AviTag™ (Avidity) (sequence GLNDIFEAQKIEWHE) and constructs were cloned between the HaloTag at the N-terminal and the 6-histidine tag next to the AviTag™ at the C-terminal. The P881G sequence was synthesised by GeneArt and inserted into the BspEI-NheI sites to create the mutant protein. Plasmids were transformed into T7 competent cells (New England Biolabs), which carried a BirA plasmid for *in vivo* biotin labelling of proteins.

Cells were grown in LB supplemented with 100 mg/ml ampicillin at 37°C. After reaching an OD_600_ of ~0.6, cultures were induced with 1 mM IPTG and 50 μM Biotin (Invitrogen) and grown at 20°C for 16 hours. Cells were resuspended in 50 mM NaPi at pH 7.0 with 300 mM NaCl, 10% glycerol supplemented with 100 mg/ml lysozyme, 5 μg/ml DNase, 5 μg/ml RNase and 10 mM MgCl_2_ and incubated on ice for 30 minutes. The lysate was passed through a French Press (G. Heinemann), then incubated with HisPur Cobalt resin (Thermo Fisher Scientific) and the His-tagged protein eluted. This procedure was followed by gel filtration using a Superdex 200 10/300 GL column (Cytiva). Proteins were stored in gel filtration buffer 10 mM HEPES pH 7.2, 150 mM NaCl, 10% glycerol and 1 mM EDTA at -80°C.

#### Human full-length vinculin expression and purification

Full-length vinculin cDNA was amplified (using the forward 5’-ATATATGCTCTTCTAGTATGCCAGTGTTTC-3’, and the reverse 5’-TATATAGCTCTTCATGCCTGGTACCAGGG-3’ primers) of 1066 amino acids long, and cloned into a pFBXNH3 insect cell expression vector containing an N-terminal His tag followed by a 3C protease cleavage site, using the fragment exchange (FX) cloning strategy (Geertsma and Dutzler, 2011). Protein expression was performed by generating a recombinant baculovirus for ExpiSf9 insect cell infection at a density of 2.0 x 106 ml^-1^ using the Bac-to-Bac system (Invitrogen). Briefly, the recombinant vinculin encoding plasmid pFBXNH3 was transformed into DH10Bac E. coli cells, which enabled the transposition of the recombinant gene into the bacmid genome. The recombinant bacmid DNA was then isolated and transfected into ExpiSf9 cells to generate the full-length vinculin-expressing baculovirus.

ExpiSf9 cells were infected with the full-length vinculin-expressing baculovirus for recombinant protein expression as described (Boujemaa-Paterski, 2020). Briefly, expressing cells were harvested by gentle centrifugation and lysed by sonication in 20 mM Tris-HCl pH 7.5, 0.4 M NaCl, 5% Glycerol, 1 mM DTT, 0.1% Triton, and protease inhibitors. Full-length vinculin was purified from clarified cell extract using a Ni-Sepharose 6 Fast Flow metal affinity chromatography, HisTrapFF (Cytiva Lifesciences). The protein was further purified on a Superdex 200 size exclusion column (Cytiva Lifesciences) and eluted with 20 mM Tris-HCl pH 7.8, 0.15 M KCl, 1 mM MgCl_2_ and 5 % Glycerol. Protein aliquots supplemented with 50% glycerol were stored at -20°C.

### Single-molecule magnetic tweezers experiments

The single-molecule experiments were conducted on a custom-made setup, shown in Fig. S1. The instrument consists of a homemade inverted microscope for tracking the reference and paramagnetic beads, and a mounting piece (inset, Fig. S1) to accurately position a magnetic tape head (Brush Industries, 902836) to apply calibrated forces. The inverted microscope uses a white LED as the light source (Thorlabs, MCWJL5-C4-6500 K), filtered with a green bandpass filter (Thorlabs, FB530-10) to improve image definition and reduce ROS production. The light is focused on a 160 mm oil-immersion objective (Zeiss, 100x Plan Apo f=160 mm) mounted on a high-precision piezo-positioner (P-725.CDD PIFOC, PI), and the image is collected with a CMOS camera (Ximea, MG013MG-ON), allowing for a working frame rate of up to 1,600 Hz.

The custom-designed mounting piece is manufactured with high-precision CNC with a tolerance <1 μm, which permits to accurately position the magnetic tape head at 450 μm from the surface and 300 μm from the magnetic beads when using #1 bottom coverslips (150 μm thick). This allows applying forces between 0 and 42 pN when using currents between 0 and 1,000 mA. The magnetic tape head is maintained under feedback with a custom-made PID current clamp circuit powered by a pair of 12 V batteries. The fluid chamber is clamped to a custom-made fork mounted on an XY-stage (Newport, MS-125-XY) allowing XY displacement in the bead-searching process. The whole instrument is mounted on a 95 mm optical rail (Thorlabs, XT95-500) and optical rail carriages (Newport, M-CXL95-80).

Single-molecule experiments are conducted in custom-made fluid chambers composed by two cover slides (40x24 mm and 22x22 mm, thickness #1, Ted Pella) sandwiched between a laser-cut parafilm pattern. The bottom glasses are cleaned by sonication on a three-step protocol: i) 1% Hellmanex at 50°C (30 min; ii) acetone (30 min); iii) ethanol (30 min), and then activated by plasma cleaning for 20 min to be silanized (immersion in 0.1% v/v 3-(aminopropyl)trimethoxysilane in ethanol for 30 min). Finally, the bottom cover slides are cured at 100°C for >30 min. The top cover slides are cleaned on 1% Hellmanex at 50 °C, for 30 min, and immersed in a repel silane solution (Cytiva, PlusOne Repel-Silane) for 30 min. The fluid chambers are assembled by melting the parafilm intercalator in a hot plate at 100°C. After assembly, the chambers are incubated in a glutaraldehyde solution (glutaraldehyde grade I 70%, 0.001% v/v) for 1 h, followed by incubation with 0.002% w/v polystyrene beads (~2.5 μm diameter, Spherotech) for 20 min, and incubated with a 20 μg/ml solution of the HaloTag amine (O4) ligand (Promega) overnight. Finally, the chambers are passivated with a BSA-sulfhydryl blocked buffer (20 mM Tris-HCL pH 7.3, 150 mM NaCl, 2 mM MgCl_2_, 1% w/v sulfhydryl blocked BSA) for >3 h. The R3^IVVI^ polyprotein construct is freshly diluted in PBS at ~2 nM and incubated for ~30 min to achieve HaloTag binding. Finally, streptavidin-coated superparamagnetic beads (Dynabeads M270, Invitrogen) are added to bind with the biotinylated polyprotein terminus for (~1 min). All experiments are performed on PBS buffer with 10 mM ascorbic acid (pH 7.3) on a temperature-controlled room at 22 ± 1°C.

### Fluctuation analysis and structure assignation

For the fluctuation analysis, we use the raw magnetic tweezers recordings (not smoothed), acquired at frame rates between 1,000 and 1,500 Hz. For a given trajectory, we first identify the visited states (***i-vi***) and extract those fragments of the recording. We then apply a threshold algorithm to systematically identify the measured signal corresponding to each conformation and remove the transition paths between conformations. For states ***iii*** and ***iv***, there is only a single conformation (**I** and **H** respectively), states ***i, ii*,** and ***vi*** have two possible conformations (**N** and **U**for ***i*** and ***ii***, **M_1_** and **M_2_** for ***vi***) and state ***v*** has three different conformations (**L_1_**, **L_2_**, and **L_3_**).

Briefly, we measure the average extension of conformations **N** and **U** (we illustrate our method with the native folding dynamics ***i***, Fig. S7A), which gives us the threshold extensions for these conformations *z_N_* and *z_U_*. Based on these thresholds, we label each extension value *z*(*t_i_*) (being *t_i_* the time marks in the measured recording) as belonging to either the **N** or **U** states or the transition paths **TP**. This is simply done by defining the transition as the fragments of trajectory where the protein escapes the folded basin (*z(t_i_)>z_N_*)to directly reach the unfolded basin (*z(t_i_)>z_U_*), and similarly for transitions from **U** to **N**. Once the transition paths have been extracted, the fragments of trajectories corresponding to states **N** and **U** are unambiguously identified (Fig. S7Bx). With this algorithm, the recording is now separated into *n* fragments in state **N** (*z_N,j_(t)*, with *j*=1, …, *n*) and *n* fragments in state ***U*** (z_*U,j*_*(t_i_)*, with *j*=1, …, *n*), being *n* the total number of transitions between **N** and **U**. The transition paths are neglected since their contribution to the molecular is spurious as they represent a diffusive excursion over a free energy barrier.

For each fragment *j*, we calculate its relative extension as *Δz_U,j_*=<*z_U,j_(t_i_)*>-z_*N*_ and its relative fluctuations as *Δσ^2^_U,j_*=(<*z_U,j_(t_i_)^2^)*>-<*z_U,j_(t_i_)>^2^)*-<*σ^2^_N_*> being <*σ^2^_N_*> the average fluctuations of state **N** (and accordingly for **N**, but its relative extension and fluctuations are trivially 0). With this procedure (Fig. S7C), we calculate the distributions of *Δz_U,j_* and *Δσ^2^_U,j_* and estimate the average change in contour length of state **U** as

$$\text{Δ}L_{C,U}=\frac{\langle \text{Δ}\sigma_{U}^{2}\rangle }{l_{K}\left[1-\coth^{2}\left(\frac{Fl_{K}}{kT}\right)+{\left(\frac{kT}{Fl_{K}}\right)}^{2}\right]}$$

Where *l_K_=1.1 nm* is the Kuhn length, and we take *kT=4.11 pN.nm*. For the unfolded conformation **U** used here as an example, we obtain *ΔL_C,U_=42.5±5.2 nm*, in agreement with the expected contour length change of R3^IVVI^ (110 aminoacids, means an approximate total contour length of 44 nm, minus the size of the folded protein). For each state, we define the fraction of unstructured sequence (or contour length) by normalizing by the *ΔL_C,U_*. For state ***v***, we adapt the described method for a three-state system.

### Detection of cis-trans isomerization of Proline 881 in R3^IVVI^

The trans-state of R3^IVVI^ is characterized by a lower mechanical stability compared to the native cis-state. In a constant force experiment, it appears as a sudden shift of the folding equilibrium towards the unfolded conformation. To systematically characterize the trans-state over long magnetic tweezers recordings, we measure the unfolded fraction using a running window as *P_U_=<t_U_>/(<t_U_>+<t_F_>)*, where <t_U_> and <t_F_> are the average times in the unfolded and folded state, respectively, as measured over a window of *n* transitions, which we take as *n*=10. The trans-state is detected as an abrupt change in *P_U_* (see Fig. S4).

## Extended Data

**Extended Data Fig. 1:**
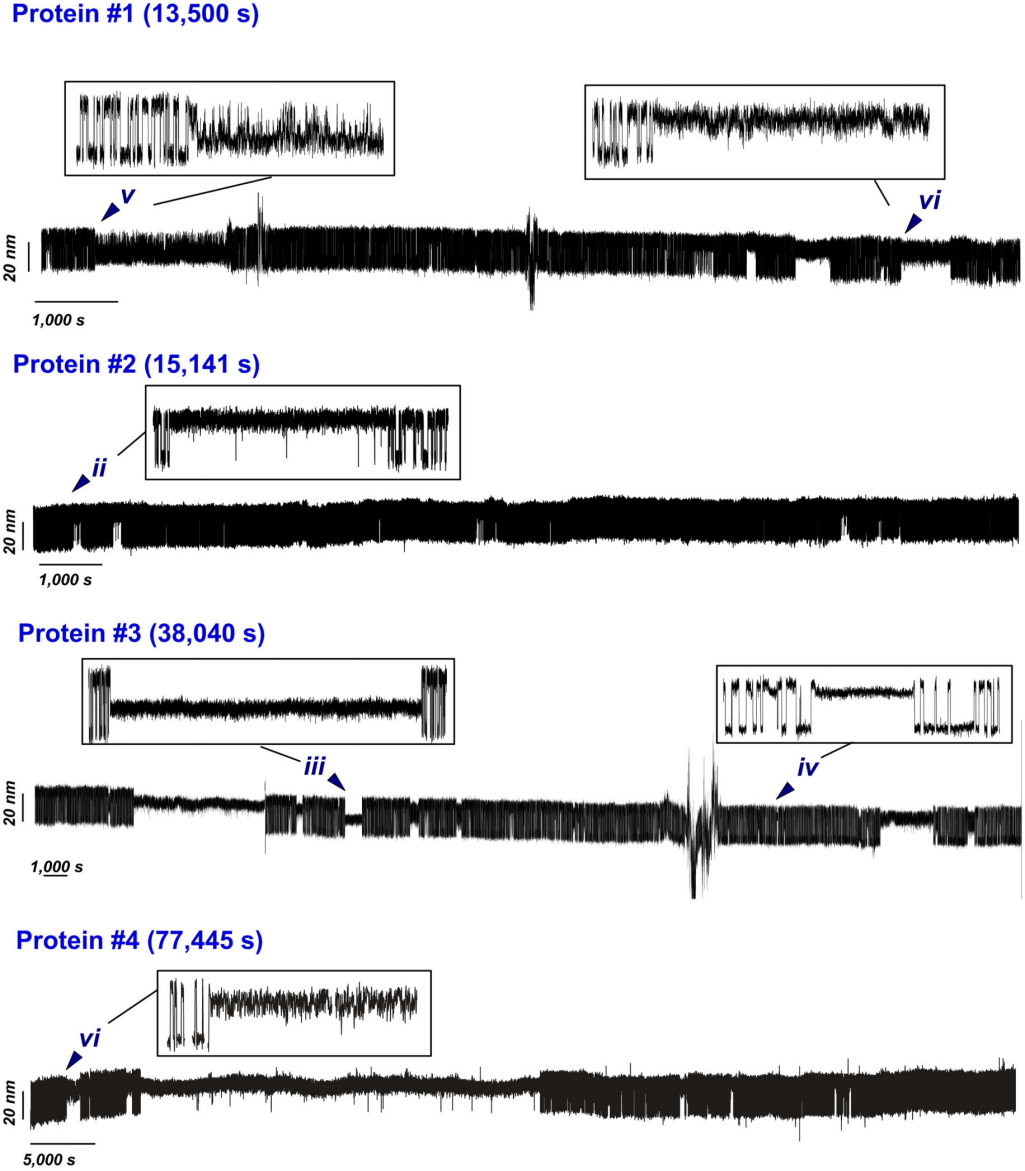
Gallery of selected magnetic tweezers recordings of R3^IVVI^ at *F*_0.5_. Each protein spontaneously visits the different identified states (i-vi), highlighted in the insets. The recordings are smoothed with a 101-points Savitzky-Golay algorithm, and unedited (no drift correction). The occasional spikes correspond to floating debris that interferes with the image analysis of the reference or the magnetic bead, but do not affect the intrinsic folding dynamics of the protein.

**Extended Data Fig. 2:**
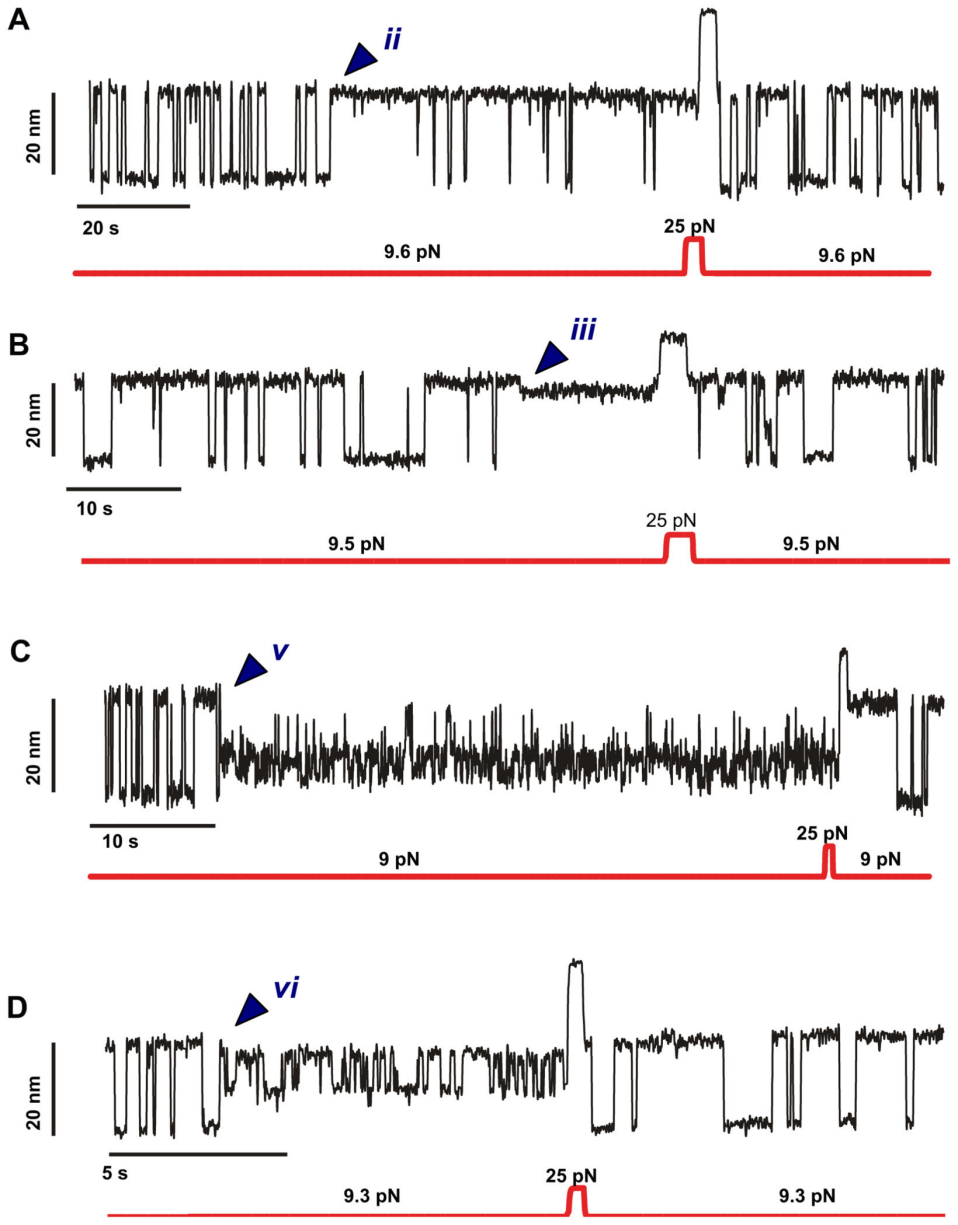
Gallery of magnetic tweezers recordings showing that a high force pulse rescues R3^IVVI^ from each trapped state back to its native folding dynamics. State **(iii)** could not be probed, as it is too short-lived to be rescued with force.

**Extended Data Fig. 3:**
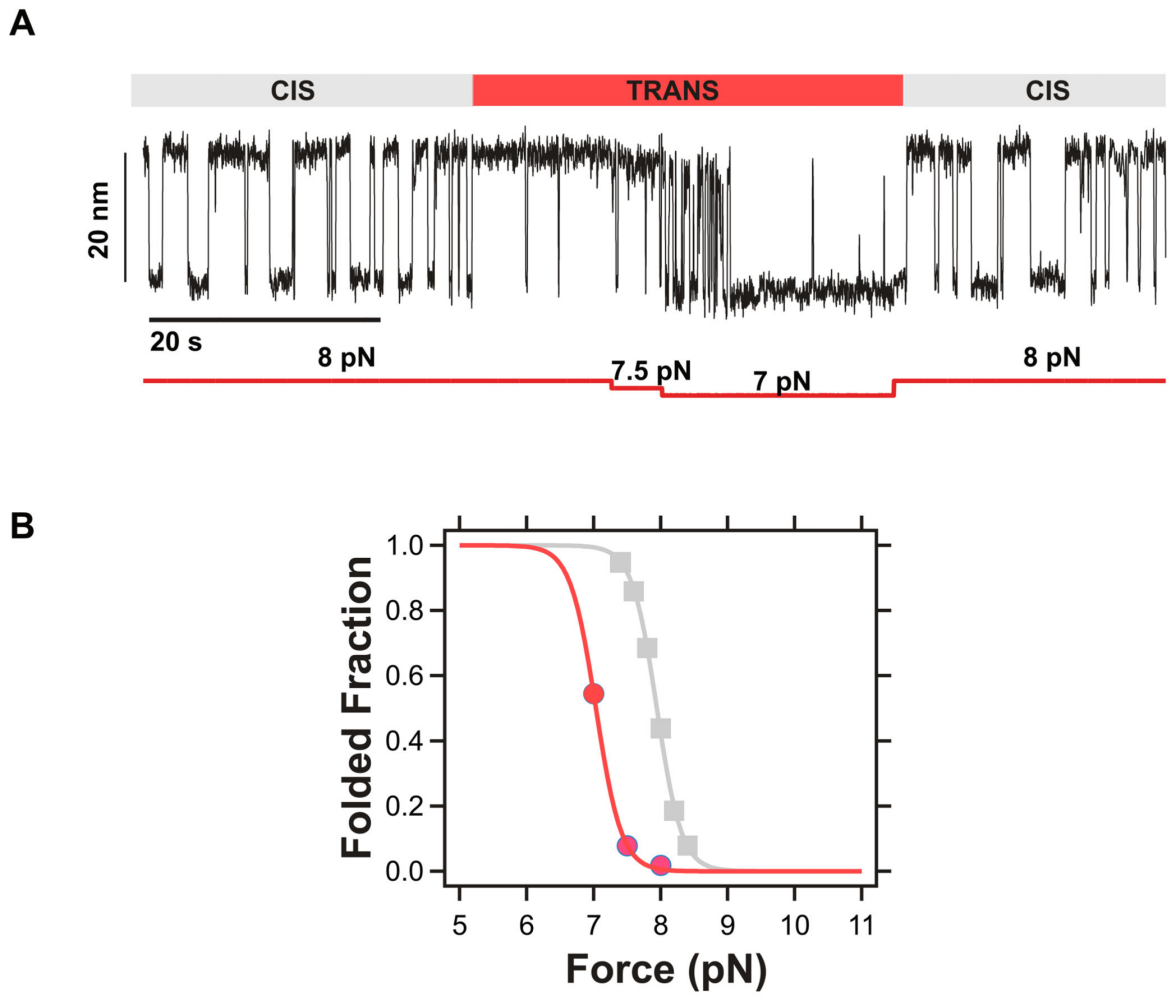
Force dependency of R3^IVVI^ with Pro^881^ in the trans state (ii). **(A)** Dynamics of R3^IVVI^ at *F*_0.5_ undergoing a spontaneous *cis-trans* isomerization of P881. The *trans*-state is probed at three different forces, showing two-state dynamics with a lower mechanical stability and faster folding/unfolding kinetics. After a few seconds, the *cis*-state is spontaneously recovered, and R3^IVVI^ goes back to its native folding dynamics. **(B)** Population of the folded form in the *cis*-state (grey) and *trans*-state (red). *Cis-trans* isomerization of Pro881 shifts the mechanical stability of R3^IVVI^ by ~1 pN.

**Extended Data Fig. 4:**
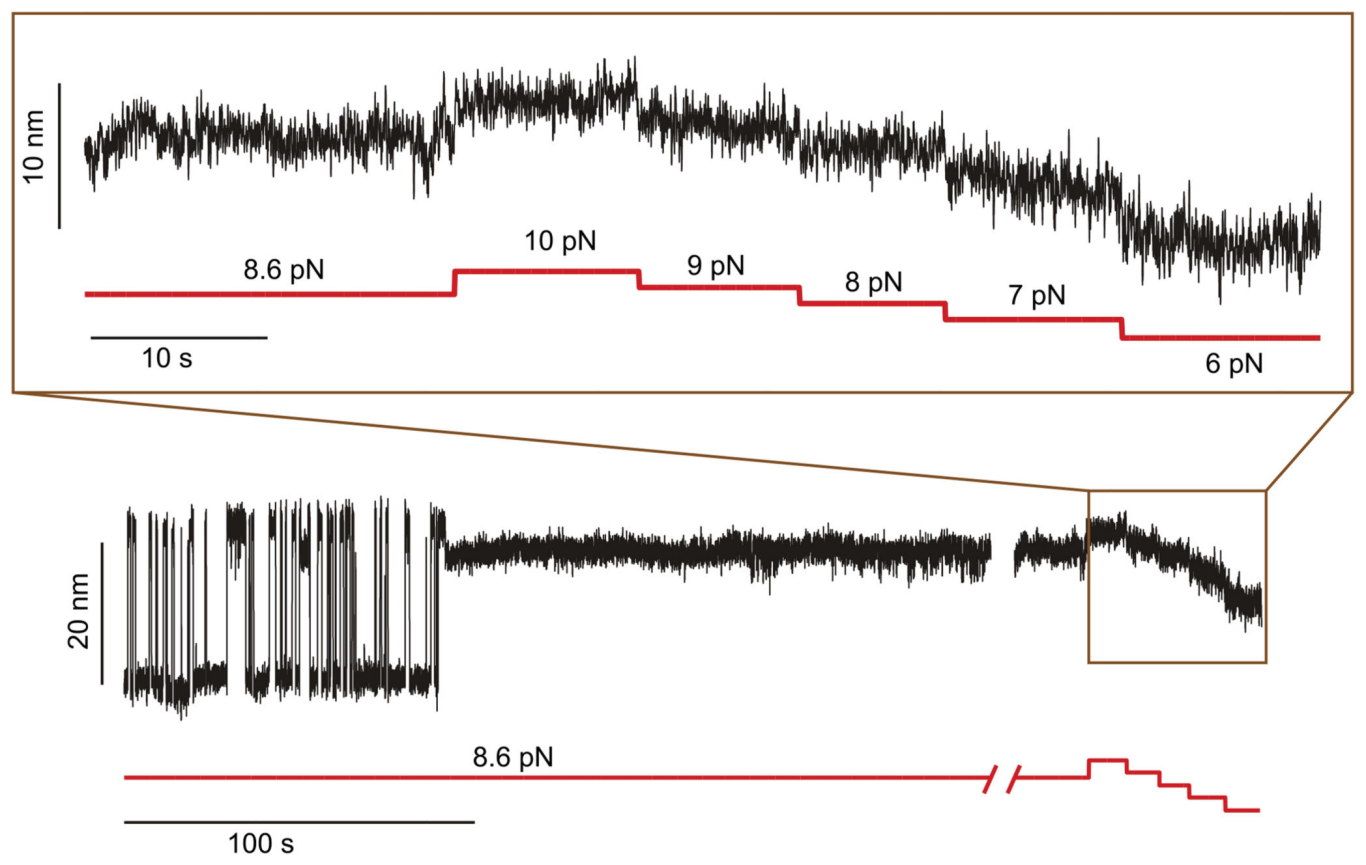
Force dependency of state (iv). R3^IVVI^ spontaneously falls into state (iv), characterized by a shorter end-to-end extension (~3 nm shorter than the unfolded state). Exploring forces between 6 and 10 pN shows that this state lacks any internal dynamics (inset).

**Extended Data Fig. 5:**
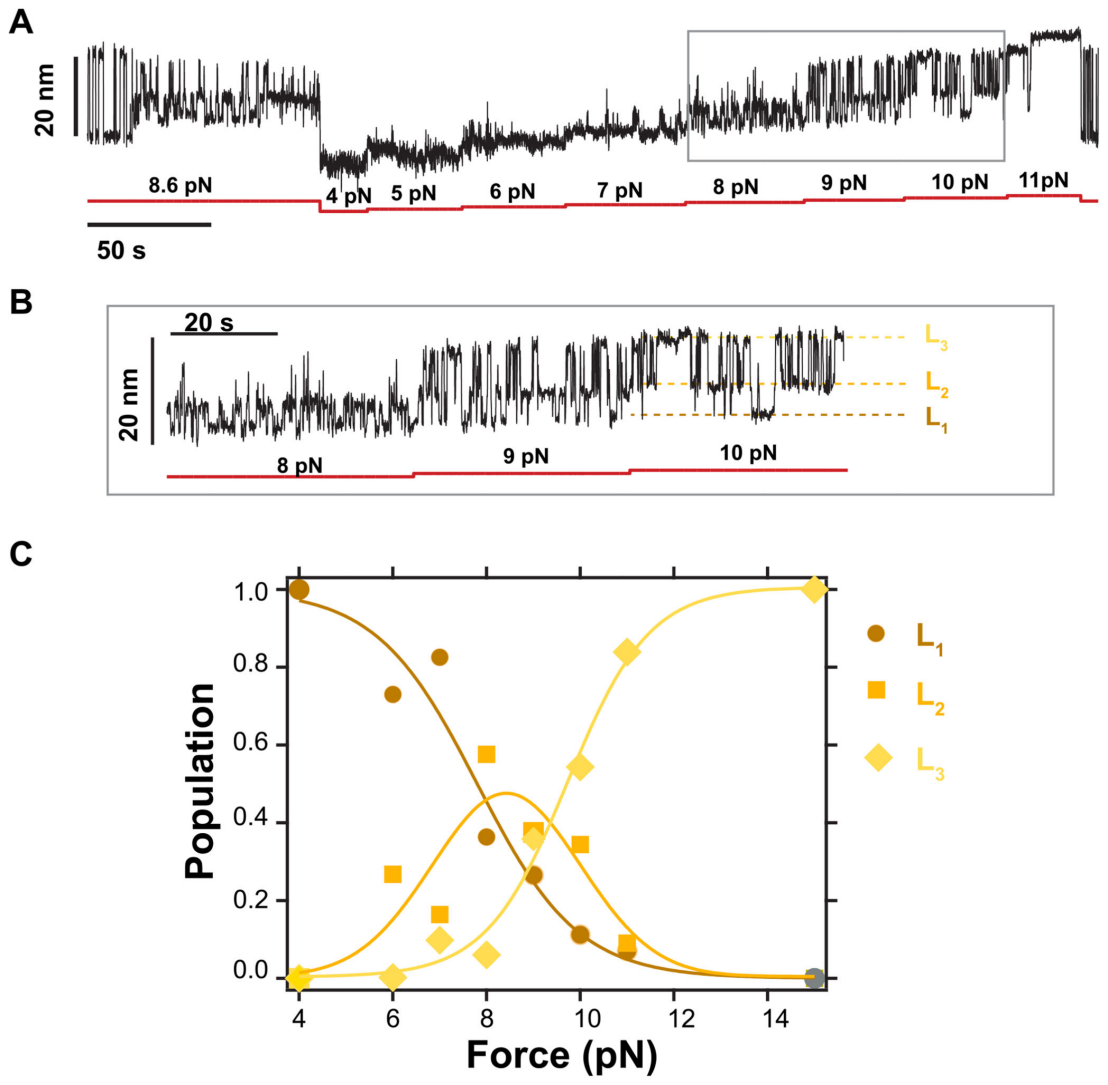
Force dependency of state (v). **(A)** Magnetic tweezers recording of R3^IVVI^ spontaneously falling into state (v). Subsequently, the dynamics of state (v) are measured between 4 pN and 11 pN, allowing one to monitor the relative population of the three conformations. **(B)** Detail of the recording in (A), which shows the three conformations, L_1_, L_2_, and L_3_. **(C)** Relative population of conformations L_1_, L_2_, and L_3_ as a function of force. Compared to the canonical native folding dynamics of R3^IVVI^ (i) which exhibits a very sharp force dependency, state **(v)** shows dynamic transitions between 5 pN and 11 pN.

**Extended Data Fig. 6:**
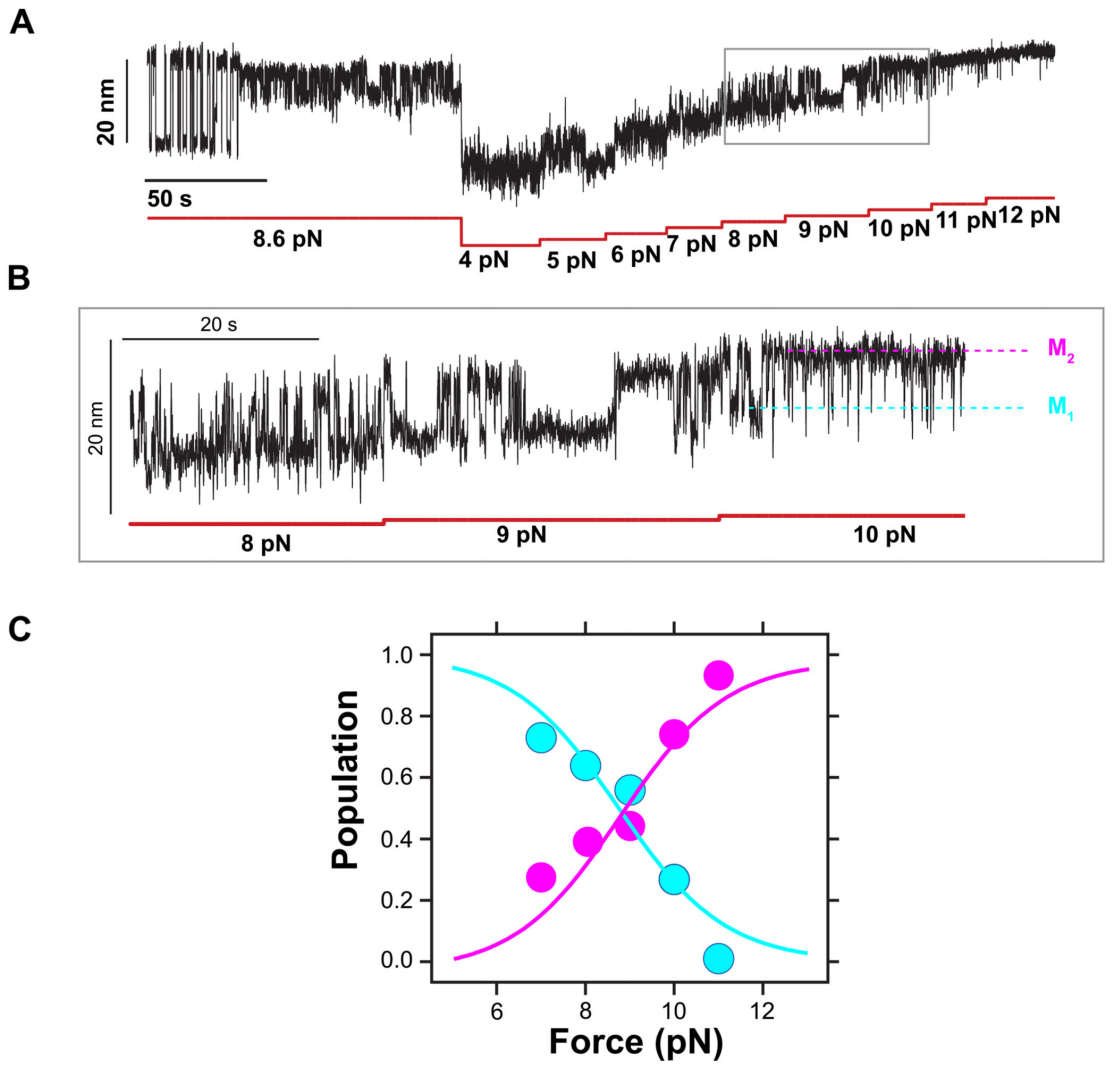
Force dependency of state (vi). **(A)** Magnetic tweezers recording of R3^IVVI^ spontaneously falling into state (vi). Subsequently, the dynamics of state (vi) are measured between 4 pN and 12 pN, allowing us to monitor the relative population of the three conformations. **(B)** Detail of the recording in (A), which shows the transitions between conformations M_1_ and M_2_. **(C)** Relative population of conformations, M_1_ and M_2_, as a function of force.

**Extended Data Fig. 7:**
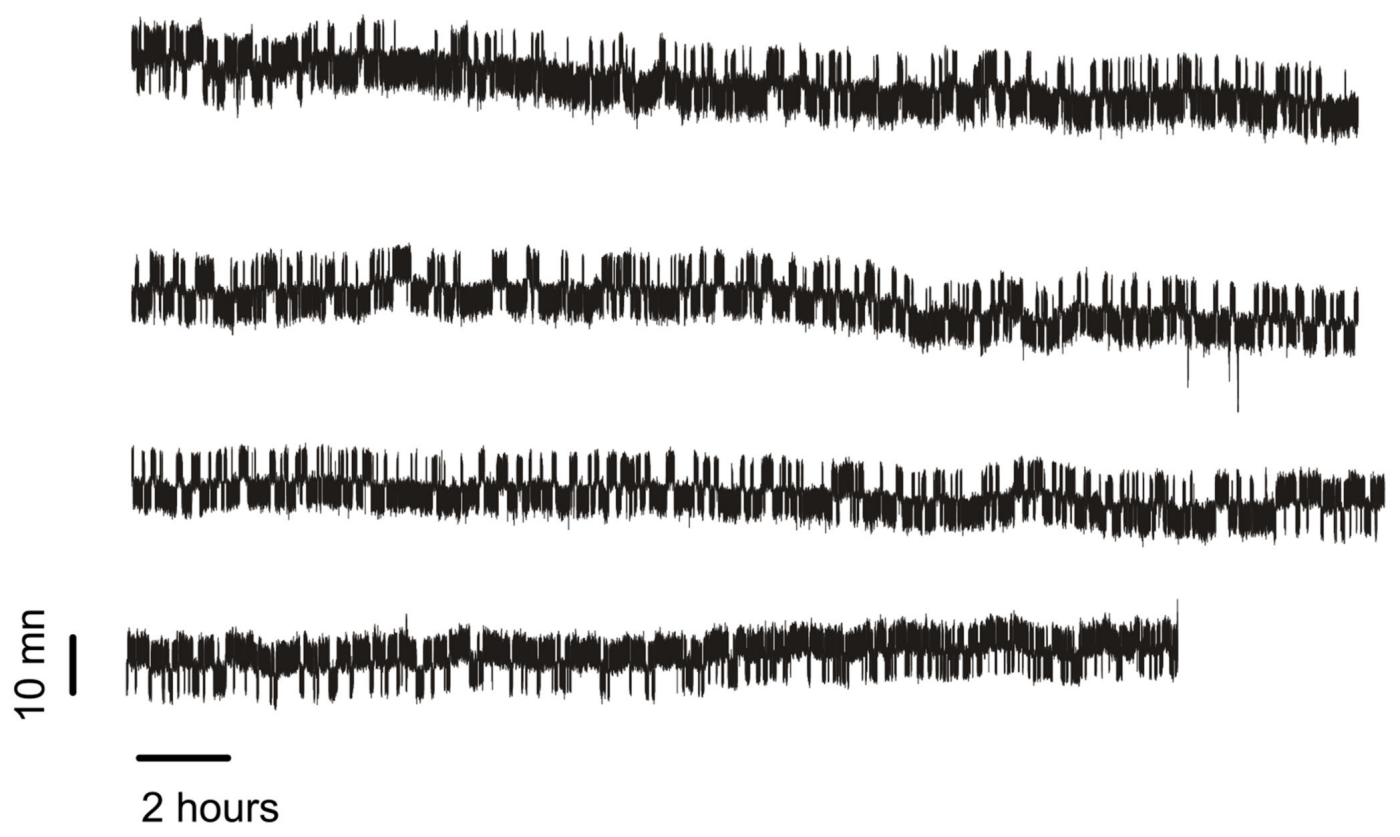
Unedited 58 hours-long recording of a single protein L at 7.4 pN. Protein L transitions in equilibrium between the folded and unfolded conformations in a two-state manner, without showing any off-pathway state.

**Extended Data Fig. 8:**
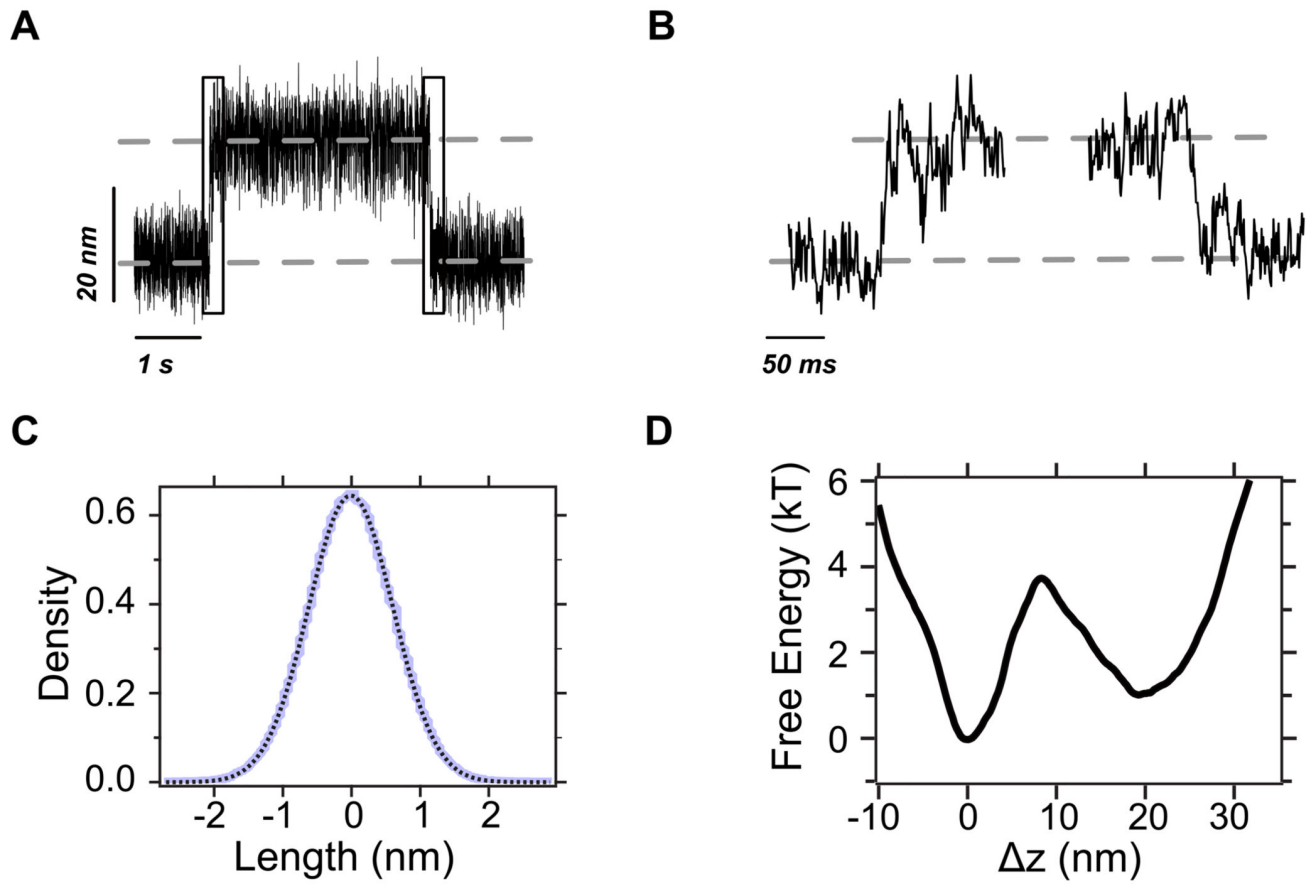
The native (un)folding dynamics in state (i) is reminiscent of a two-state behaviour with our instrumental time resolution. **(A)** Raw magnetic tweezers recording showing native unfolding and refolding transitions within state (i) (squared regions). **(B)** Detail of a native unfolding (left) and refolding (right) transition path, sampled at ~1,500 Hz. **(C)** Histogram of the trajectory of the reference bead after filtering low-frequency components arising from local drift (blue line). Being firmly attached to the surface, the spread in the vertical position of the reference bead arises mostly due to artifacts in the image analysis algorithm that influence the experimental estimation of the molecular extension. The data are well-fit by a Gaussian (black dotted line) with *σ=0.85nm*, which we use as the point-spread function to reconstruct the free energy landscape following a deconvolution procedure. **(D)** Free energy landscape of talin R3^IVVI^ in state (i) at *F*_0.5_. The free energy landscape was reconstructed from >10,000 native (un)folding transitions following a deconvolution procedure (Jansson algorithm, Woodside et al., *Science*, 2006) using a point-spread function estimated from the trajectory of the reference bead (panel C). The dynamics within state (i) are two-state, as the landscape displays two free energy basins with a single free energy barrier of ~4*kT* separating them.

**Extended Data Table. 1:** Conformational properties of the different configurational states of the talin R3^IVVI^ domain. All quantities are calculated taking the folded conformation (**F**) as reference. The unstructured contour length (*L_c_*) is calculated from the variance, assuming the freely jointed chain with a Kuhn length *l*_K_ = 1.1 nm and normalizing by the contour length of the unfolded conformation (42.5±5.2 nm). Errors are standard deviation.

*State*	*Conformation*	*Quantity*	*Value*
***i***	**F**	Δ*z* (nm)	0.0
		Δσ^2^ (nm^2^)	0.0
		Unstructured *L_c_* (nm)	0.0
		Unstructured *L*_c_ (%)	0.0
	**U**	Δ*z* (nm)	18.2±0.7
		Δσ^2^ (nm^2^)	5.8±0.7
		Unstructured *L*_c_ (nm)	42.5±5.2
		Unstructured *L*_c_ (%)	100
***iii***	**I**	*Δz*(nm)	5.4±0.5
		Δσ^2^ (nm^2^)	0.16±0.1
		Unstructured *L*_c_ (nm)	1.3±0.7
		Unstructured *L*_c_ (%)	5.5±0.7
***iv***	**H**	Δ*z* (nm)	15.6±0.3
		Δσ^2^	2.8±0.4
		Unstructured *L*_c_ (nm)	22.6±3.0
		Unstructured *L*_c_ (%)	63±6.9
***v***	**L_1_**	Δ*z* (nm)	3.3±0.5
		Δσ^2^ (nm^2^)	0.6±0.7
		Unstructured *L*_c_ (nm)	4.4±5.1
		Unstructured *L*_c_ (%)	10±12
	**L_2_**	Δ*z* (nm)	7.6±0.7
		Δσ^2^ (nm^2^)	3.4±0.7
		Unstructured *L*_c_ (nm)	24.9±5.1
		Unstructured *L*_c_ (%)	58.6±12
	**L_3_**	Δ*z* (nm)	14.9±0.6
		Δσ^2^ (nm^2^)	4.6±0.6
		Unstructured *L*_c_ (nm)	33.7±4.4
		Unstructured *L*_c_ (%)	79.3±10
***vi***	**M_1_**	Δ*z* (nm)	7.45±0.2
		Δσ^2^ (nm^2^)	2.8±0.4
		Unstructured *L*_c_ (nm)	26.7±1.9
		Unstructured *L*_c_ (%)	53±8.9
	**M_2_**	Δ*z* (nm)	14.3±0.1
		Δσ^2^ (nm^2^)	2.8±0.4
		Unstructured *L*_c_ (nm)	25.2±5.7
		Unstructured *L*_c_ (%)	59±14

**Extended Data Table. 2:** Kinetic analysis of average transition times between the different conformations. All events included in this kinetic analysis are from recordings with a minimal duration of at least 10,000 s at *F*_0.5_. Errors are standard error of the mean.

*State*	*Transition*	*Average Transition Time (s)*	*Number of Events*
***i***	**F ➔ U**	1.4±0.1	522,721
	**U ➔F**	1.3±0.1	522,721
***ii***	**U (*i*) ➔U (*ii*)**	1,372.5±100.2	152
	**F ➔U (*ii*)**	0.07±0.10	1,504
	**U ➔F (*ii*)**	3.2±0.2	1,505
	**U (*ii*) ➔U (*i*)**	63.4±8.7	154
** *iii* **	**U➔I**	6,135±3,500	11
	**F ➔I**	12,504±6,054	2
	**I ➔U**	7.8±0.3	12
	**I ➔F**	6.5±5.4	4
***iv***	**U ➔H**	1,251.8±140.3	108
	**H ➔U**	96.3±12.3	108
***v***	**U ➔L_3_**	2,153.4±310.6	57
	**L_3_ ➔L_2_**	0.09±0.01	1,985
	**L_2_ ➔L_1_**	0.42±0.01	12,298
	**L_1_➔L_2_**	0.37±0.01	12,298
	**L_2_➔L_3_**	2.52±0.11	1985
	**L_3_ ➔U**	270.4±790.8	57
***vi***	**U ➔M_2_**	1,744±211	79
	**M_1_ ➔M_2_**	0.91±0.02	15,980
	**M_2_ ➔M_1_**	0.25±0.03	15,981
	**M_2_ ➔U**	122.0±8.3	79

## Supplementary Material

Fig. S1

## Figures and Tables

**Figure 1:**
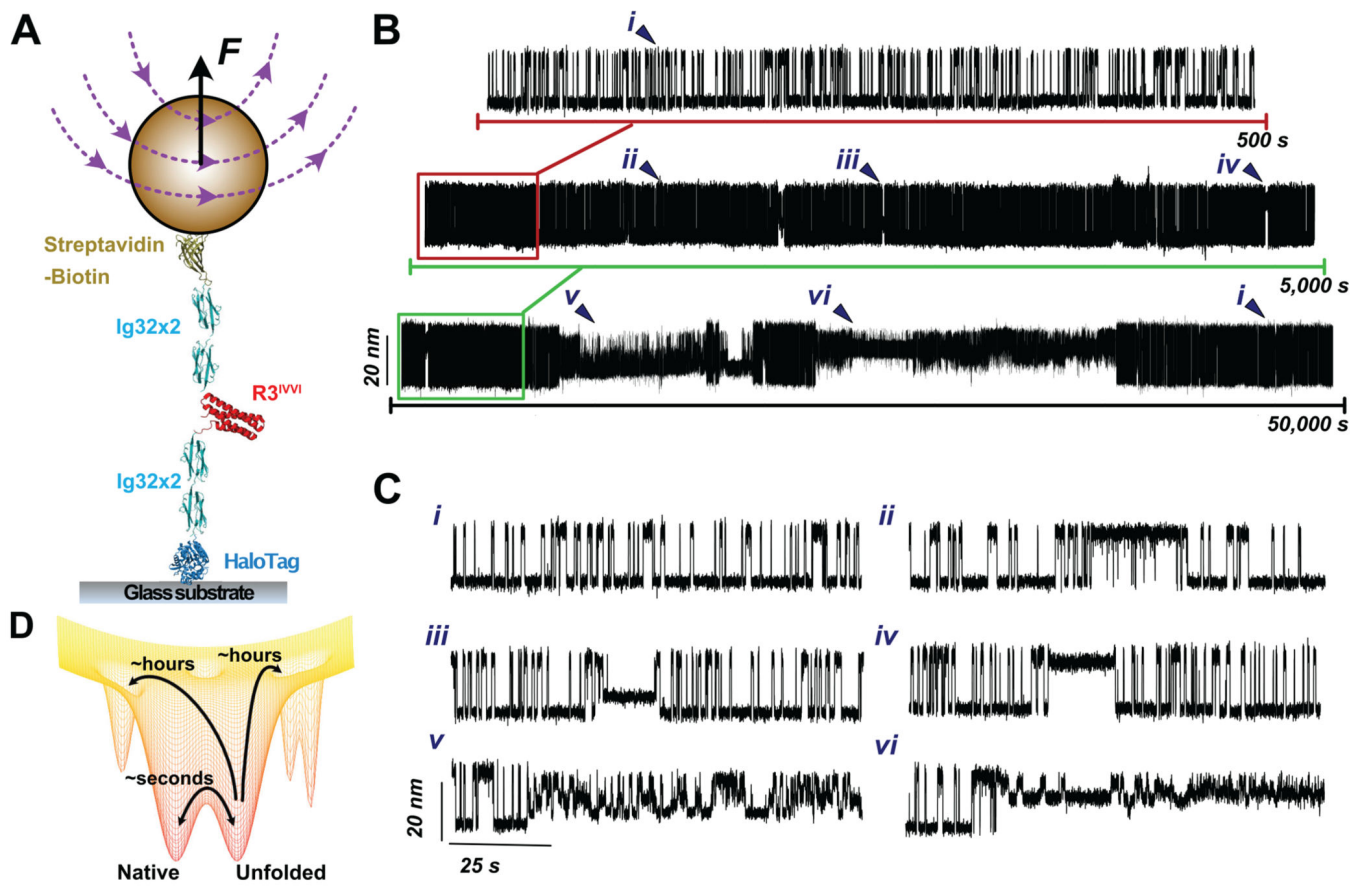
Enhanced experimental sampling of R3^IVVI^’s conformational space reveals low-probability states. **(A)** Schematics of our magnetic tweezers assay for measuring the equilibrium dynamics of the talin R3^IVVI^ domain under force. R3^IVVI^ is flanked by two mechanically stiff titin Ig32 domains serving as molecular handles. A C-terminus HaloTag allows covalent anchoring to a functionalized glass surface and an N-terminus biotinylated AviTag tethers the protein construct to an M270 streptavidin superparamagnetic bead. **(B)** Magnetic tweezers trajectory of R3^IVVI^ at the coexistence force *F*_0_._5_ (here 8.5 pN). Over a timescale of a few minutes (upper), R3^IVVI^ transitions in equilibrium between the folded and unfolded protein forms in an all-or-none fashion. When the measuring time is significantly increased to a few (middle) or several (lower) hours, R3^IVVI^ reveals a much more complex conformational space, populated by six distinct states (represented by roman numerals ***ii***-***vi***) including the native state (***i***). **(C)** 100-second detail of the six different states that form the full conformational space of the talin R3^IVVI^ domain. **(D)** Cartoon representation of a conformationally-rich folding free energy landscape. Over short timescales, the protein samples a local equilibrium involving the native and unfolded conformations. However, if the measuring time is significantly expanded, previously inaccessible regions separated by high energy barriers can be quantitatively explored.

**Figure 2:**
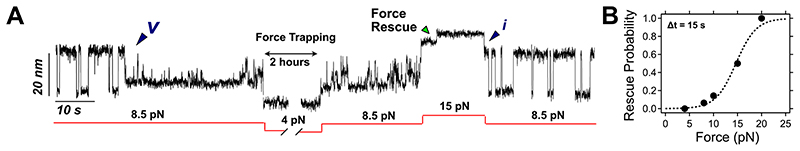
Mechanical force rescues R3^IVVI^ from the rare, low probability states by bringing it back to its native folding dynamics. **(A)** Magnetic tweezers recording showing the effect of mechanical force on R3^IVVI^ rare states. Initially, R3^IVVI^ transitions in a two-state fashion between the natively folded and unfolded forms to spontaneously fall into state ***v***. By decreasing the force down to 4 pN, R3^IVVI^ is trapped for an extended period of time (here ~2 hours). Subsequently, a high force pulse of 15 pN applied for 15 seconds drives R3^IVVI^ to its unfolded conformation, and the protein is rescued back to its native state (***i***), hopping between the native and unfolded protein forms at 8.5 pN. **(B)** Probability of rescuing R3^IVVI^ folding dynamics over a 15 seconds-long pulse at different forces. Data measured on *N>3* molecules, with >10 events per force (error bars: s.e.m.).

**Figure 3:**
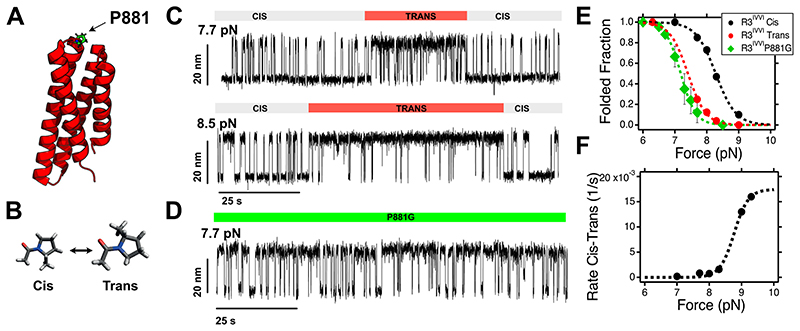
A proline switch regulates the mechanical response of talin R3^IVVI^ (state ***ii***). **(A)** Structural representation of talin R3^IVVI^ highlighting the position of P881 in the loop between helices #3 and #4. **(B)** Schematics of the cis- and trans- isomers of the proline amino acid. **(C)**Magnetic tweezers trajectories of R3^IVVI^ undergoing spontaneous cis-trans isomerization of P881, which reduces its mechanical stability. **(D)** Folding dynamics of the P881G R3^IVVI^ mutant, which recapitulates the nanomechanics of R3^IVVI^ in the trans-isomer, fingerprinted by lower mechanical stability and faster folding kinetics. **(E)** Folded fraction of the folded R3^IVVI^ in the cis-(black) and trans-(red) state, and of the P881G mutant (green), which recapitulates the folding dynamics of the R3^IVVI^ trans state (error bars: s.e.m.). **(F)** Rate of cis-trans isomerization of P881 in talin R3^IVVI^. The rate of isomerization increases with force, suggesting that the trans- form is favoured if talin R3^IVVI^ is unfolded. The force dependency can be described as *r*_t-c_(*F*)=*P*_U_(*F*)*k*_t-c_, where *P*_U_ is the unfolded fraction of talin R3^IVVI^ (inverse of the folded fraction shown in panel C, measured independently), and *k*_t-c_ is the isomerization rate of P881, assumed to be force-independent. From the fit, we obtain *k*_t-c_= 0.0017 s^-1^ (~ 588 s) (error bars: s.e.m.).

**Figure 4:**
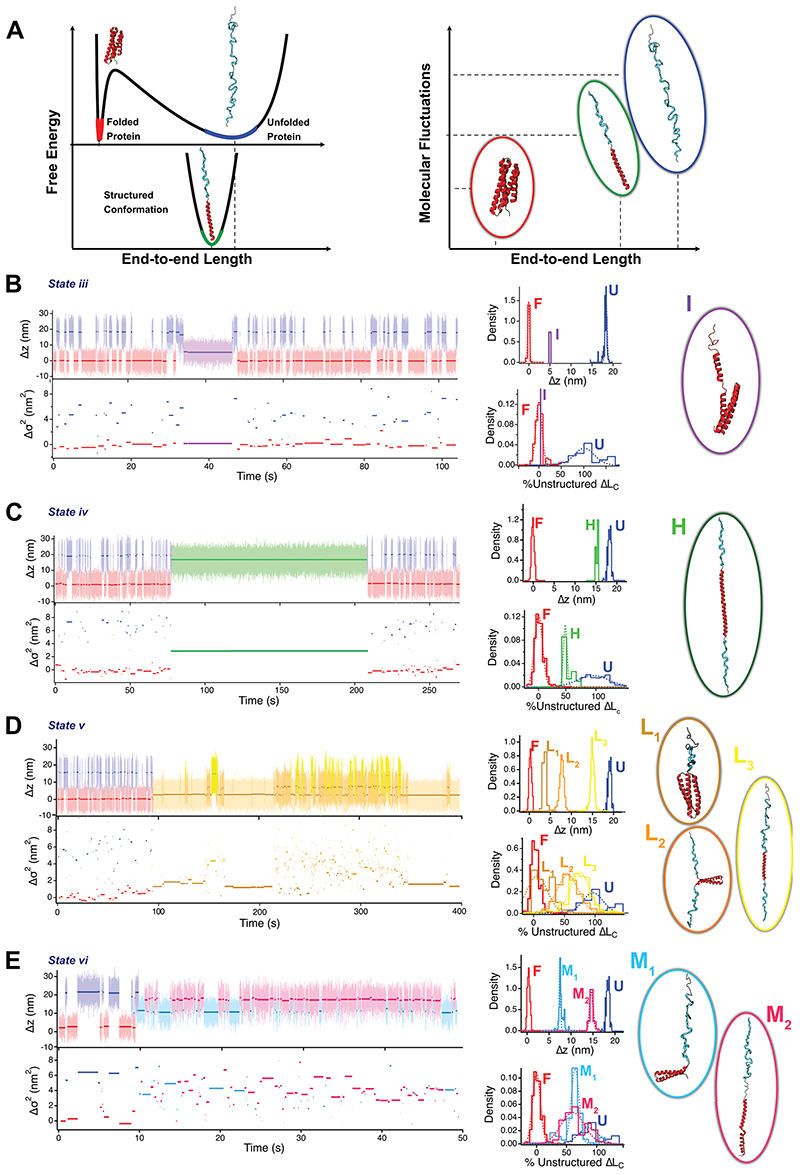
Fluctuation analysis of R3^IVVI^ dynamics fingerprints its wide conformational repertoire, enabling to suggest potential compatible protein structures defining each rare state. **(A)** Free energy diagram of a protein under force. Upon unfolding mechanically (red to blue), the protein acquires an extended conformation, greatly increasing its average end-to-end length and molecular fluctuations, which is reflected in a wider free-energy basin. If the protein acquires a new conformation composed of both structured (red) and unstructured (blue) segments, its end-to-end length will only be slightly shorter than that of the unfolded state, but its fluctuations will be greatly reduced, given that only the unstructured sequence (blue) contributes to the measured fluctuations. Hence, the end-to-end length and molecular fluctuations can be combined to fingerprint protein conformations. (*Left upper*, from **B** to **E**) Magnetic tweezers trajectories of R3^IVVI^ dynamics spontaneously falling on states ***iii*** (**B**), ***iv*** (**C**), ***v*** (**D**) and ***vi*** (**E**). Individual conformations are characterized by a color and capital letter, different from the states, labelled by roman numerals. (*Left lower*) Change in variance Δσi^2^ of each measured conformation. (*Central*) Distributions of end-to-end length (upper histograms) and fraction of unstructured sequence calculated from Δσi^2^ assuming *l*_k_=1.1 nm and normalizing by the contour length increment of R3^IVVI^ (Δ*L*_c_=42.5 nm). (*Right*) Potential structural models of each of the conformations visited by R3^IVVI^, compatible with the measured end-to-end changes and molecular fluctuations, although other structures are certainly possible. In each case, structured segments are represented in red while unstructured segments are represented in blue.

**Figure 5:**
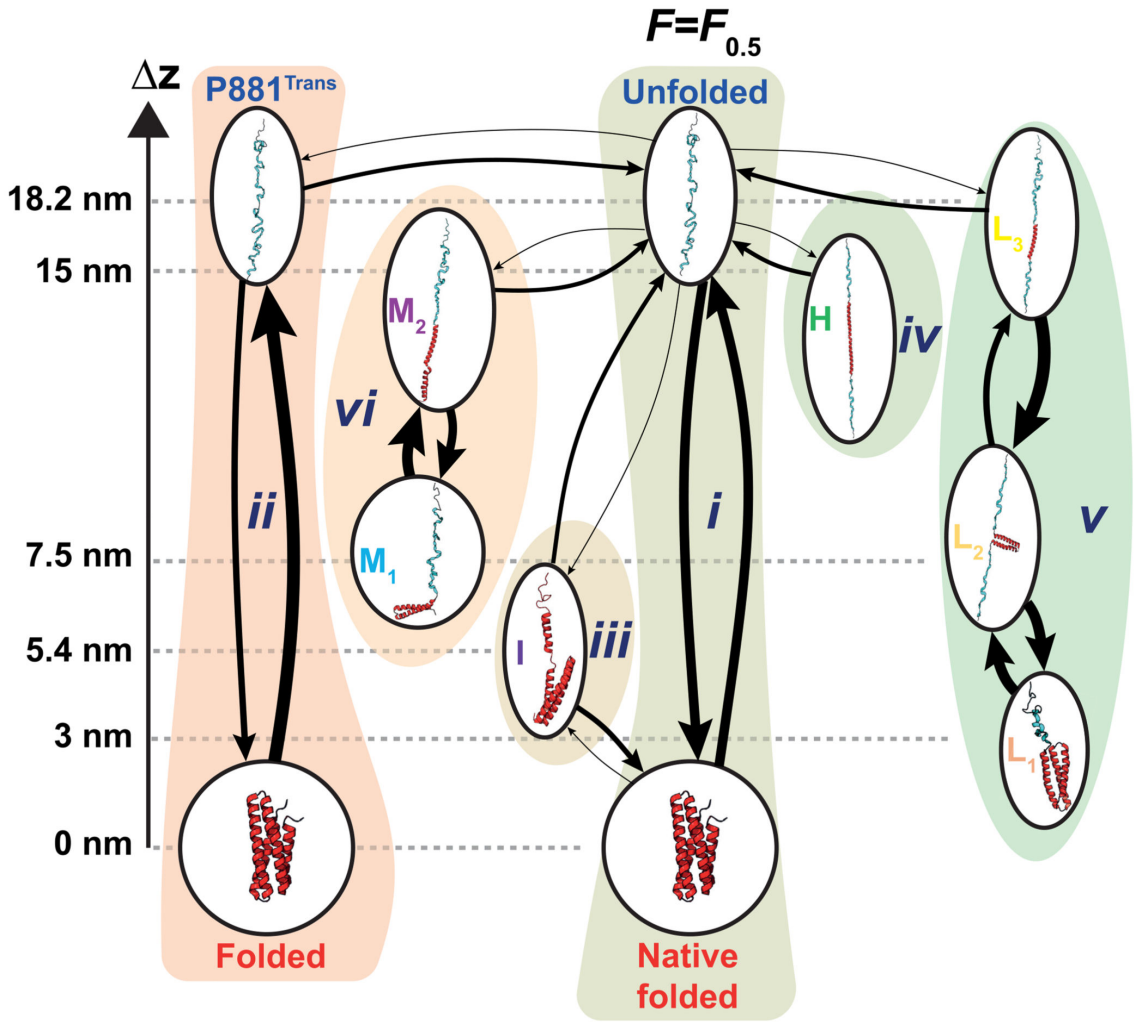
Conformational space of the talin R3^IVVI^ domain represented as a kinetic network measured at *F*_0.5_. Arrows indicate observed transitions between specific configurations, characterized by the forward and backwards average transition times. For each transition (arrow) the thickness of the line is proportional to the transition probability from/to a given conformation, normalized to the unfolded protein form. Note the distinction between states (labelled by roman numerals), and conformations within states (labelled by capital letters). Some states (namely ***i***, ***ii*, *v*** and ***vi***) exhibit multiple conformations with internal dynamics between them, while others (***iii*** and ***iv***) show a single conformation. Potential suggested structures compatible with our analysis are included. Data measured from *N* = 13 molecules accumulating a total measuring time of 144 hours (see Table S2).

**Figure 6:**
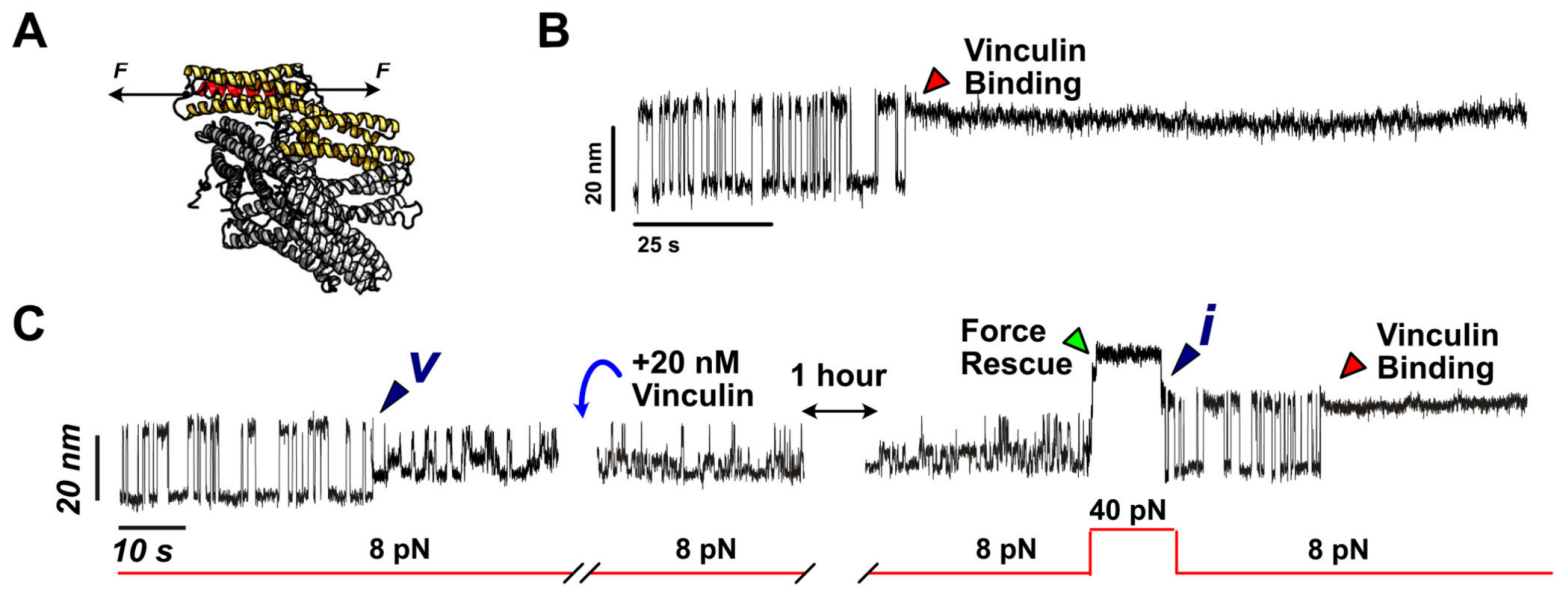
Probing full-length vinculin’s ability to bind R3^IVVI^’s rare states. **(A)** Schematics of full-length vinculin binding to a vinculin binding site under force. The vinculin head domain 1 (Vd1, which is the talin-binding domain) is shown in yellow. **(B)** Fingerprint of vinculin binding to talin R3^IVVI^. Vinculin binds to unfolded talin, contracting its extension by ~3 nm and stopping its native folding dynamics. **(C)** When adding 20 nM vinculin to R3^IVVI^ trapped in state ***v***, R3^IVVI^ continues to exhibit its characteristic dynamics, indicating that vinculin cannot bind to state ***v***. After rescuing R3^IVVI^ back to its native folding dynamics, vinculin quickly binds.

## Data Availability

Source data are available for this paper. All other data that support the plots within this paper and other findings of this study are available from the corresponding author upon reasonable request.

## Code availability

The code is available from the corresponding author upon reasonable request.
