# Supplementary material for: Enhanced statistical sampling reveals microscopic complexity in the talin mechanosensor folding energy landscape
Source: Nat Phys. Author manuscript; Available in PMC 2023 Jan 18. (PMC7614079; doi:10.1038/s41567-022-01808-4)
Supplement: Fig. S1 [file EMS158115-supplement-Fig__S1.pdf]

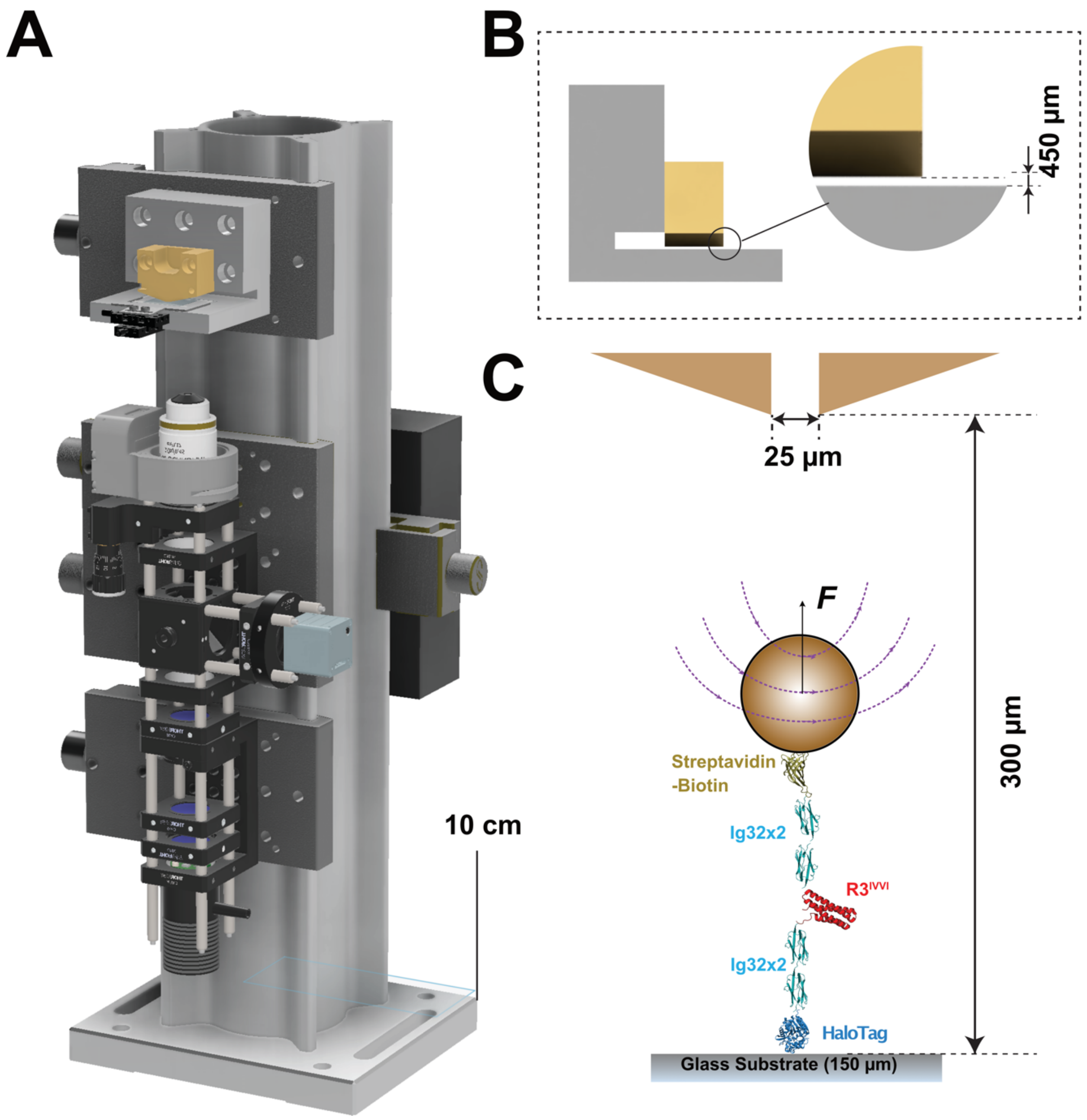

Figure S1: Magnetic tweezers design for ultra-stable single-protein measurements. (A) Three-dimensional model of our magnetic tweezers setup. **(B)** Detail of the measuring apparatus. The magnetic tape head is mounted on a high-precision CNC-manufactured piece that positions the tape head's gap at 450 µm from the surface, allowing us to apply calibrated forces by controlling only the electric current supplied to the tape head. **(C)** Schematics of our magnetic tweezers assay for measuring $R3^{IVVI}$ dynamics under force. By using #1 bottom glasses (150 µm thick), the bead is positioned 300 µm away from the tape head's gap, allowing us to apply forces between 0 and 42 pN when using currents between 0 and 1,000 mA.

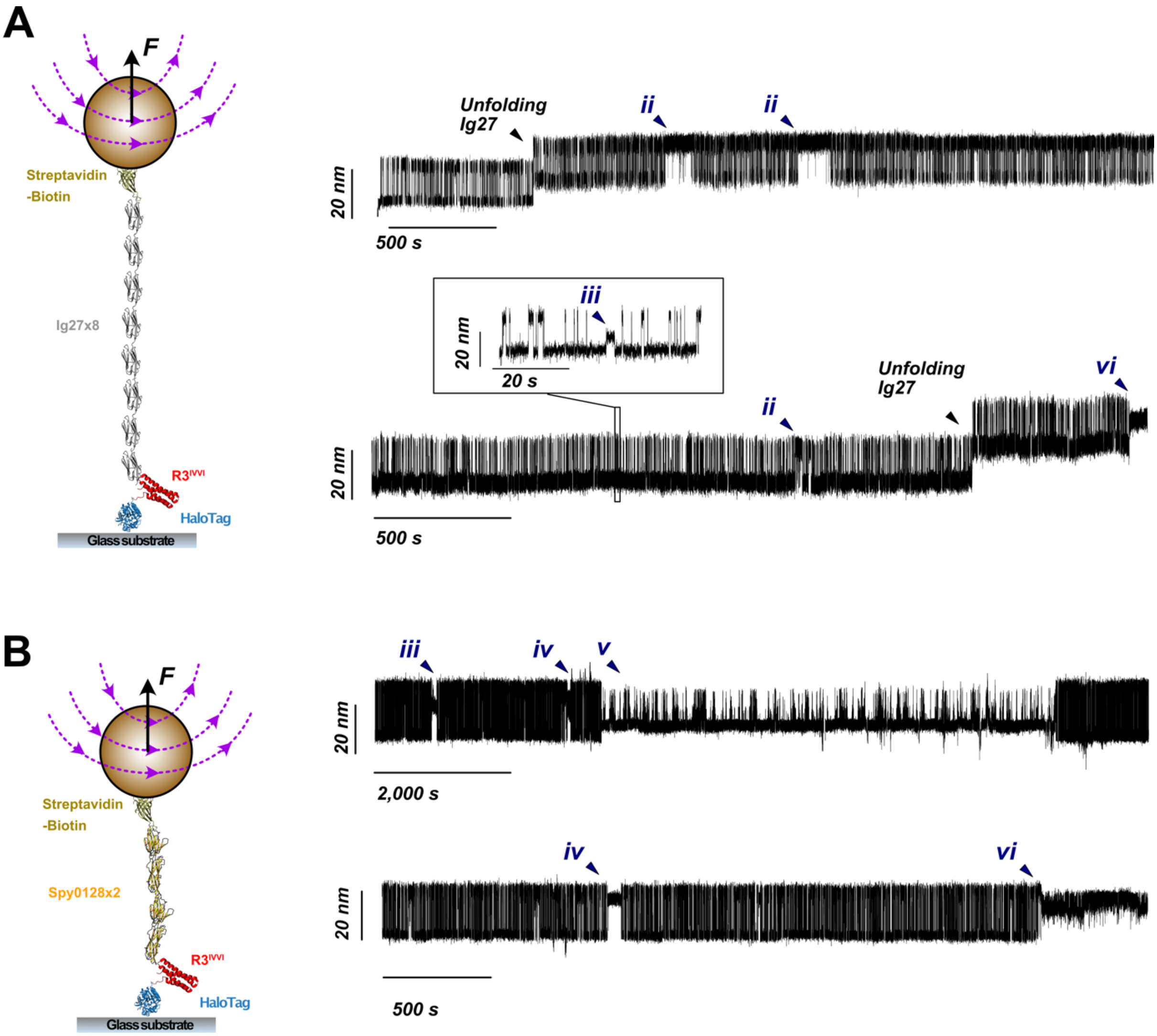

Figure S2: R3^{IVVI} low-probability states are independent of the molecular handles employed in the polyprotein construct. (A) Schematics of the magnetic tweezer assay where the R3^{IVVI} is flanked between 8 domains of the titin Ig27 and the HaloTag. Recordings using this construct at a $F_{0.5}$ display the whole repertoire of low-probability states. Due to the lower mechanical stability of Ig27 compared to Ig32, we occasionally observed the stochastic unfolding of an Ig27 domain (fingerprinted by a step-size of ~15 nm), which makes this construct not ideal for long experiments. **(B)** Schematics of the magnetic tweezer assay where the R3^{IVVI} is flanked between 2 inextensible Spy0128 domains and a HaloTag. Studying the dynamics of R3^{IVVI} at a $F_{0.5}$ enables detection of all the low-probability states measured with the Ig32 molecular handles.

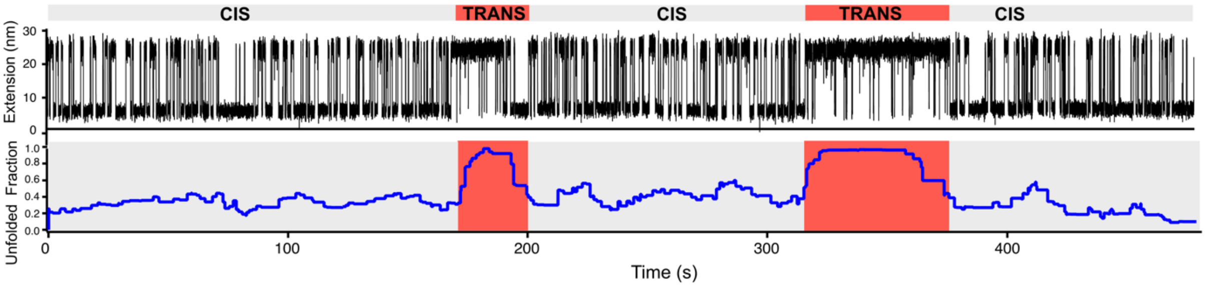

Figure S3: Illustration of the algorithm for detection of the isomerized trans-state of P881. (Upper) Typical trajectory of $R3^{IVVI}$ at $F_{0.5}$, undergoing two cis-trans isomerization events. The trans-state is characterized by lower mechanical stability, which results in a shift of the equilibrium towards the unfolded conformation. (Lower) The trans-state can be systematically detected as a sudden increase in the unfolded fraction, measured as a running window calculated over 10 transitions.

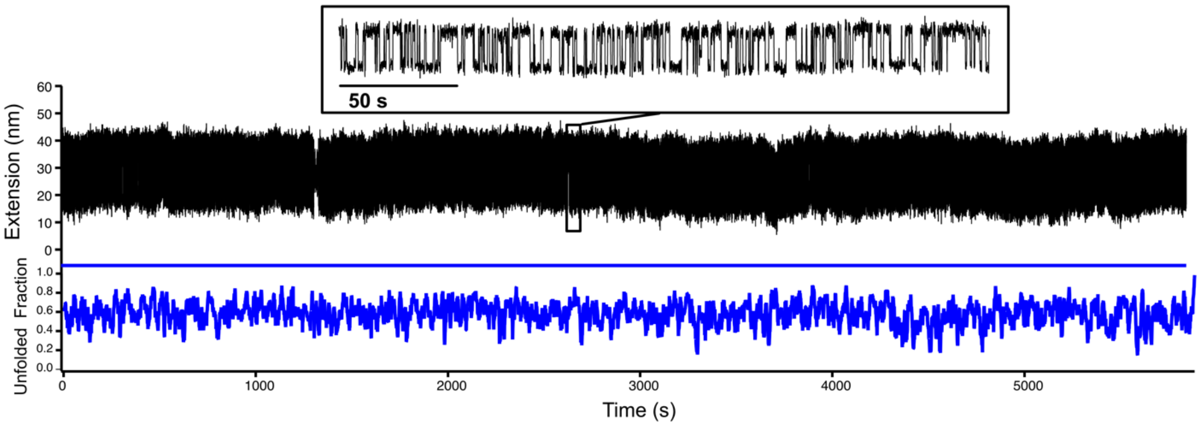

Figure S4: Magnetic tweezers recording of R3^{IVVI} P881G at 7.5 pN. Substituting Pro881 by Glycine abrogates the proline isomerized state (***ii***), which is not observed over hours-long measurements. Glycine mimics the trans-state of Proline, resulting in a protein with lower mechanical stability and faster folding dynamics.

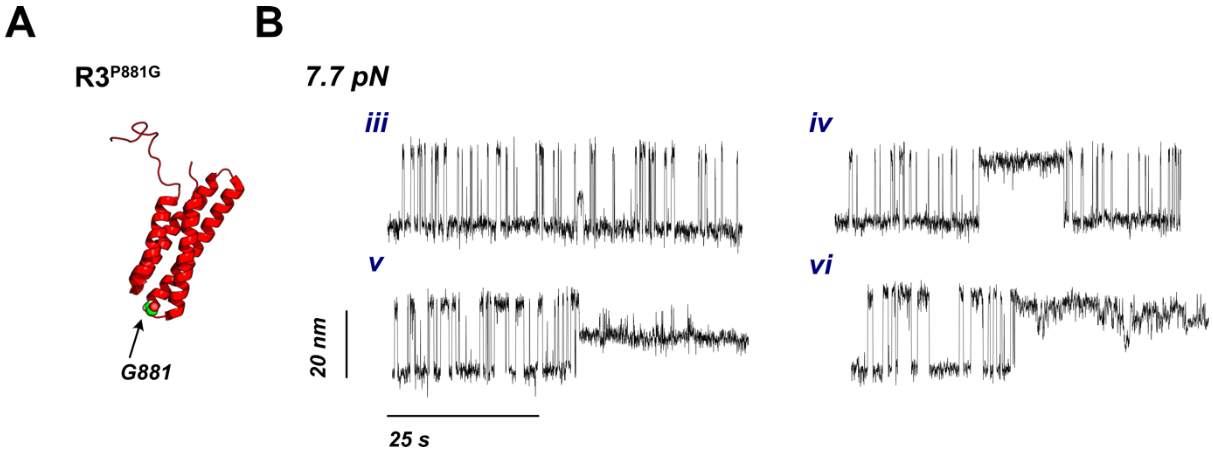

Figure S5: Talin R3^{IVVI} P881G exhibits low-probability states. (A) Structural representation of talin R3^{IVVI} highlighting the mutation of the Proline881 into a Glycine, which mimics the P881-trans state. (B) Magnetic tweezers trajectories of talin R3^{IVVI} P881G at $F_{0.5}$ displaying all low-probability states (except ***ii***).

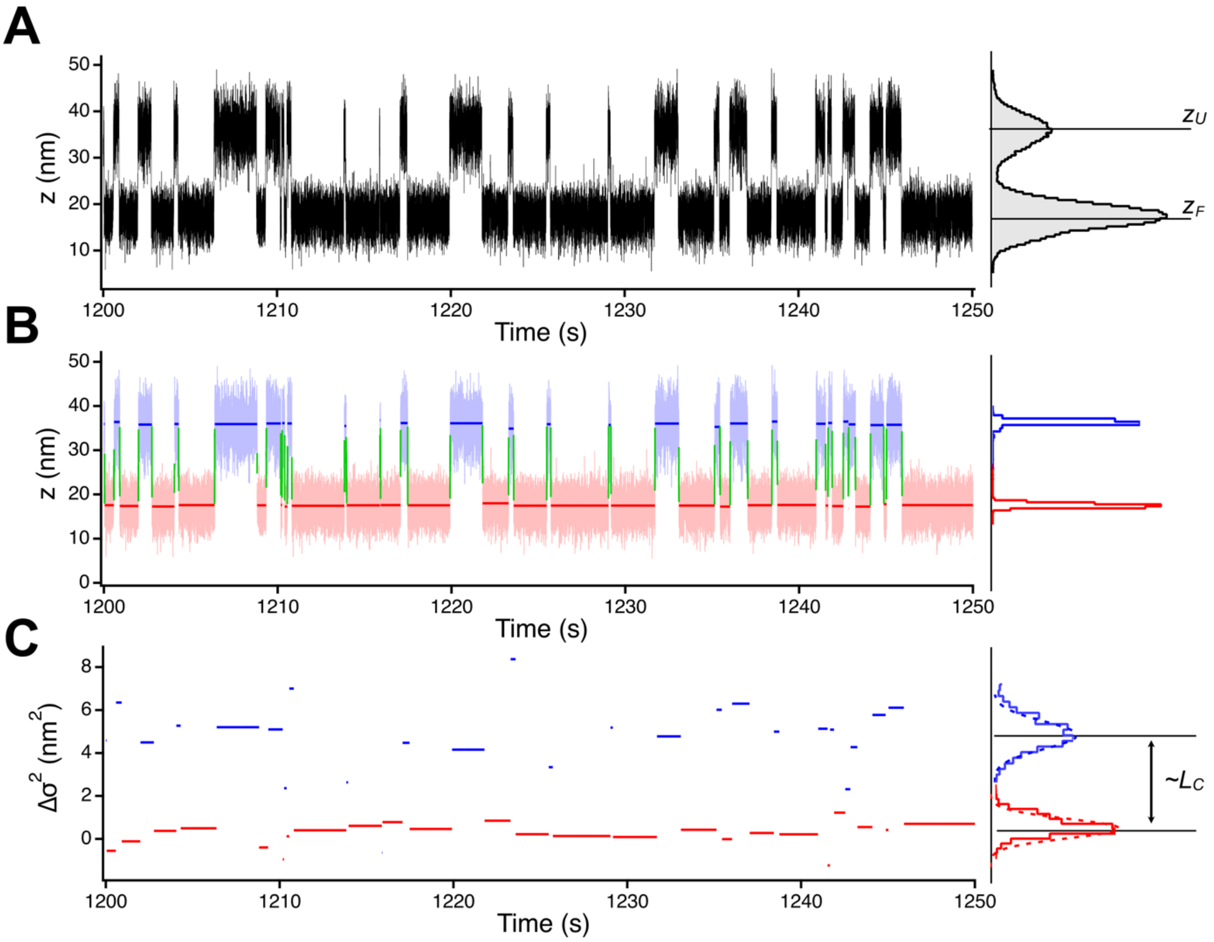

Figure S6: Analysis method for calculating the molecular fluctuations of the R3^{IVVI} conformational states, here illustrated for the native state (i). (A) Fragment of an R3^{IVVI} trajectory in state **i**. We calculate the average extensions of each conformation from the distribution of z(t) (here **F** and **U**) that establishes the thresholds for the discretization algorithm. (**B**) Processed trajectory, with conformation **F** labelled in red, conformation **U** in blue, and the transition paths in green. The average extension of each dwell fragment is shown in a darker color, and the calculated distributions in the right. **(C)** Change in variance calculated for the shown trajectory. The unfolded conformation shows higher fluctuations, which scale with a change in contour length of ~42 nm.

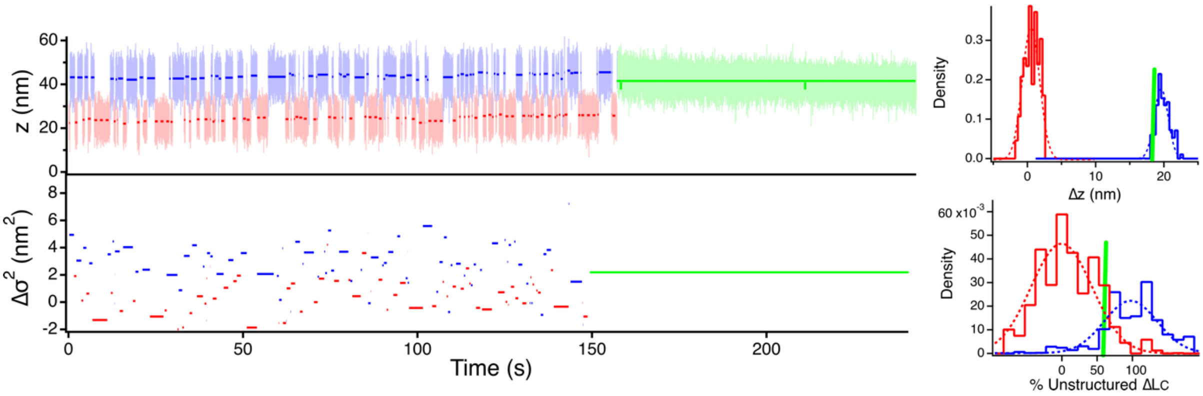

Figure S7: **Full-length vinculin binding recapitulates the molecular properties of R3^{IVVI} state *iv*.** After full-length vinculin binds (green trace), the extension of R3^{IVVI} decreases by ~3 nm, while its molecular fluctuations drop by ~50%. This indicates that half of R3^{IVVI} sequence acquires a secondary structure that does not contribute to the molecular fluctuations, likely the coil-to-helix transition previously proposed to be triggered by vinculin binding. These properties are fully equivalent to those measured for state *iv*.

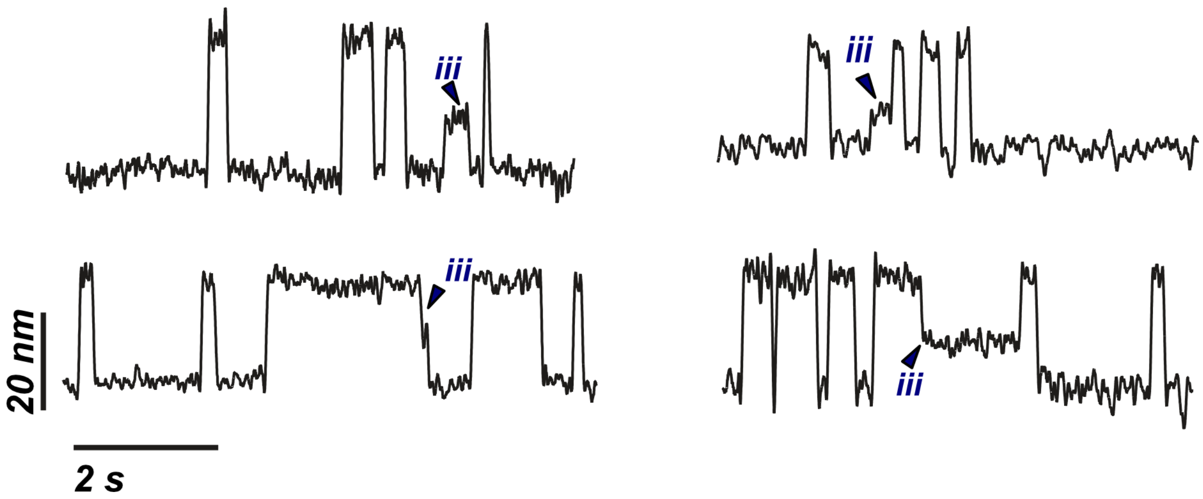

Figure S8: **State *iii* is a true folding intermediate, connected to both the folded and unfolded protein forms.** State ***iii*** can be reached from the folded (upper traces) or unfolded (lower traces) forms, and escape to the folded (left traces) or to the unfolded (right traces) protein forms.

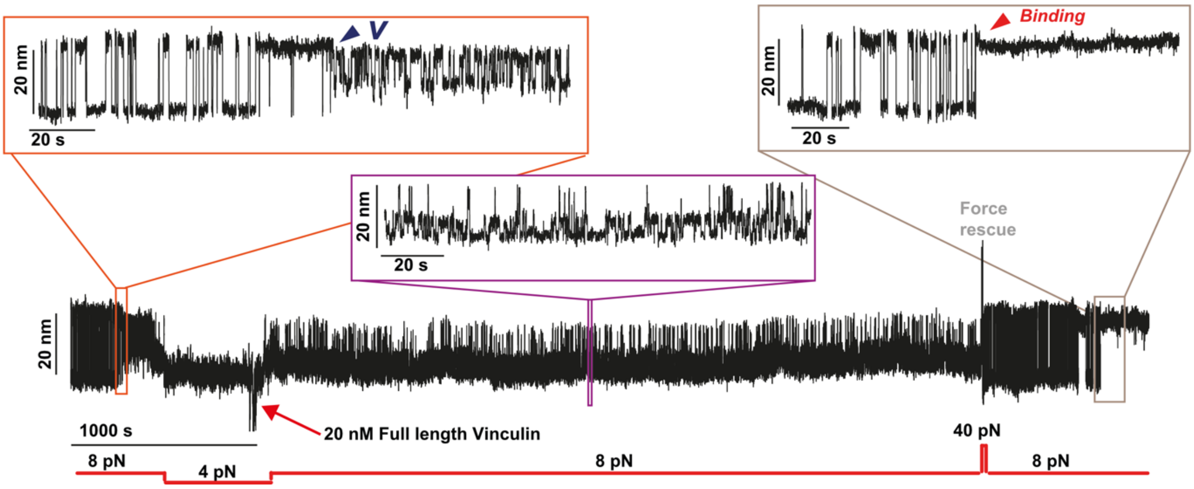

Figure S9: Full-length vinculin does not bind to state *v*. Magnetic tweezers recording showing the force protocol to demonstrate the inability of state ***v*** to recruit full-length vinculin. At 8 pN, R3^{IVVI} spontaneously falls into state ***v*** (orange inset). Subsequently, we decrease the force down to 5 pN to trap R3^{IVVI} in state ***v*** while we add 20 nM full-length vinculin (arrow). When increasing the force back to 8 pN, R3^{IVVI} shows the characteristic dynamics of state ***v*** (purple inset) for over an hour, indicating no vinculin binding. After rescuing R3^{IVVI} back to its native folding dynamics with a 40 pN pulse, full-length vinculin quickly binds after a few seconds, which stops the dynamics of R3^{IVVI} (brown inset).

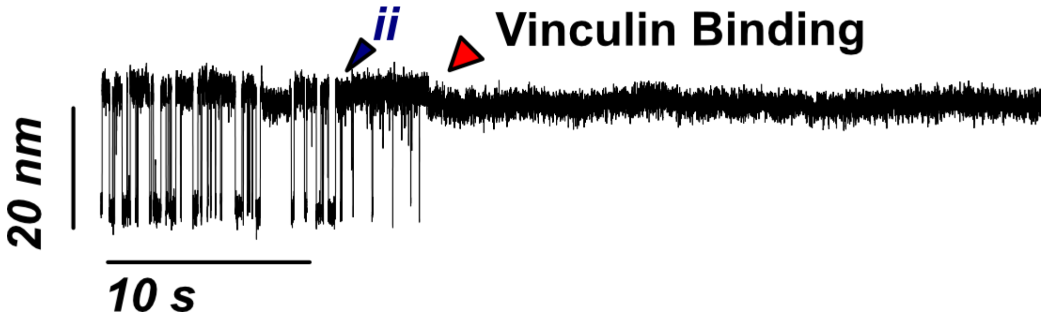

Figure S10: Full-length vinculin binds to state *ii*. This indicates that the trans-state of P881 does not interfere with full-length vinculin binding.

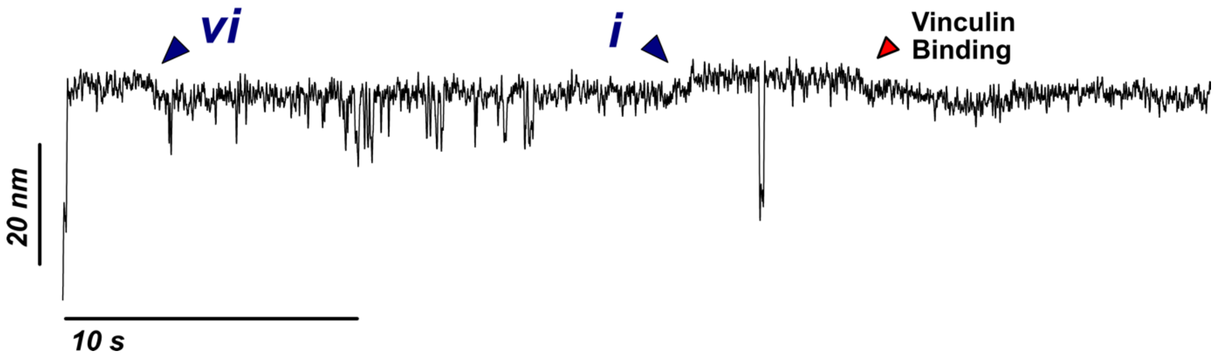

Figure S11: Full-length vinculin does not bind to state *vi*. R3^{IVVI} spontaneously falls into state ***vi*** and escapes after ~15 s, after which vinculin quickly binds.
